# Structural dynamics in the host-parasitoid system of the pine needle gall midge (*Thecodiplosis japonensis*) during invasion

**DOI:** 10.7717/peerj.3610

**Published:** 2017-08-22

**Authors:** Won Il Choi, Mun-Jang Jeon, Young-Seuk Park

**Affiliations:** 1 Division of Forest Insect Pests and Diseases, National Institute of Forest Science, Seoul, Republic of Korea; 2 Department of Forest Resources, Daegu University, Gyeongsan, Republic of Korea; 3 Department of Biology, Kyung Hee University, Seoul, Republic of Korea; 4 Department of Life and Nanopharmaceutical Sciences, Kyung Hee University, Seoul, Republic of Korea

**Keywords:** Pine needle gall midge, Invasive species, *Thecodiplosis japonensis*, Host–parasitoid, Competition, Parasitoid

## Abstract

The structural dynamics of host–parasitoid populations play a key role in the mechanism of natural community development with invasive species. Species invading new habitats experience coevolution with their newly acquired natural enemies, and their population dynamics are driven by a complex interaction between biological and environmental factors. We examined the biological and environmental factors which potentially influence a community of parasitoids throughout the 25-year invasion history of the pine needle gall midge (PNGM), *Thecodiplosis japonensis*, an important pest of pines in eastern Asia. We found that differences in establishment sequence and competitive ability among the parasitoids attacking this species determined the parasitoid community’s structure and dynamics. In particular, the timing for the initial establishment of the host–parasitoid association, incomplete superiority in competition among parasitoids, and indirect competition by a combination of the parasitoids were important factors for determining community’s structure and dynamics. Finally, the history of change in the community composition could be explained by the phenology differences in its member species, mediated by environmental factors.

## Introduction

The structural dynamics of host–parasitoid populations play a key role in the mechanism of natural community development with invasive species. After the invasion of a species in a new area, the host–parasitoid relationships are established through the coevolution of the population of the host and that of its newly acquired natural enemy. The coevolution is driven by a complex interaction between the biological and environmental factors. Most species have multiple natural enemies (Poulin, 1997; Raffel, Martin & Rohr, 2008), which compete among themselves; such competition is a potentially important mechanism of coexistence in natural communities. The coexistence of competitive species is facilitated by niche segregation based on differentiation in biological traits among the competitors, which leads to the coevolution of competitive species that structures the ecological guilds (Bonsall, Jansen & Hassell, 2004; Vayssade et al., 2012; Ghara & Borges, 2010). Phenological divergence is one of the most important biological traits underlying niche segregation (Edwards & Stachowicz, 2010; Hackett-Jones, Cobbold & White, 2009; Sachet et al., 2009) because it prevents interference competition between competitors (Amarasekare, 2002).

Environmental factors are considered a major force shaping community assembly (HilleRisLambers et al., 2012; Jiang & DeAngelis, 2013; Laughlin et al., 2012). Several abiotic factors affect the demographic rate of individuals and influence their population fitness. They also affect the interactions between populations of different species (Begon, Townsend & Harper, 2006). Changes in environmental factors such as temperature can alter host–parasitoid interactions. For example, Meisner, Harmon & Ives (2014) reported effects of temperature on long-term population dynamics in a host–parasitoid system by investigating population cycles of pea aphids and their most common parasitoid, *Aphidius ervi* (Haliday), in Wisconsin, USA, and suggested that increasing the long-term mean temperature decreases the cycle period of host–parasitoid populations, increases the cycle amplitude, and tends to destabilize pea aphid—*A. ervi* dynamics.

Biological invasions frequently occur when organisms are moved into a new place through both active or passive process (Mack et al., 2000; Novak, 2007), which results in the loss of native biodiversity and changes the community structure (Allendorf & Lundquist, 2003). Species invading new habitats often find themselves free from natural enemies, a situation occasionally leading to a population outbreak during the initial stage of the species’ invasion. Later, these species may be exposed to new natural enemies, and a new set of host–parasitoid relationships are established (Choi & Park, 2012). The invasive species then experience coevolution with their newly acquired natural enemies and their long-term population dynamics, under the influence of these new natural enemies, is driven by a complex interaction between biological and environmental factors within the ecological community.

The pine needle gall midge (PNGM), *Thecodiplosis japonensis* Uchida et Inouye (Diptera: Cecidomyiidae), is one of the most serious pests of pine trees in eastern Asia, including Korea and Japan (Ko, 1982; Soné, 1987; Chon et al., 2000; Park & Chung, 2006; Choi et al., 2011). The first occurrence of PNGM in Korea was reported in Mokpo and Seoul in 1929, possibly by independent introductions (Choi & Park, 2012; Ko, 1982). Four platygastrid species, *Inostemma matsutama* Yoshida, *Inostemma seoulis* Ko, *Inostemma hockpari* Ko*,* and *Platygaster matsutama* Yoshida*,* have been reported as parasitoids of the PNGM (Jeon et al., 2006). The life histories of *I. seoulis* and *P. matsutama* are relatively well known compared to those of *I. matsutama* and *I. hockpari* (Jeon et al., 1985; Yoshida & Hirashima, 1979). *I. seoulis* and *P. matsutama* are the dominant species of the guild in Korea, although their distributions and abundances differ in space and time (Jeon et al., 1985; Park et al., 2001). The geographical distribution of *I. seoulis* overlaped with that of PNGM, whereas *P. matsutama* appeared to be restricted to western and southern Korea. *I. matsutama* has only been observed in southern Korea (Jeon et al., 2006; Lee et al., 1985). The suppression of host population by these parasitoids was reported to be low in the early stages of the PNGM invasion but was strengthened after the midge became established (Park et al., 2001).

In this context, we hypothesize that differences in competition ability among the parasitoids, mediated by environmental factors, influence the structural dynamics of host–parasitoid systems during the invasion of the host species. To test this hypothesis, we analysed the community structure of the parasitoids and the competition between parasitoids on the host species. We also evaluated the influence of environmental factors on the community structure.

## Materials and Methods

### Study sites and sampling plan

The abundance of PNGM and its parasitoids was monitored in a pine forest in Yeongcheon, in the southern part of Korea (35°54′N, 128°51′E), dominated by Japanese red pine (*Pinus densiflora* Siebold & Zucc*.*, >50%) and pitch pine (*P. rigida* Mill., approximately 40%). To collect PNGM and its parasitoids that overwinter in the soil, twenty emergence traps (height 34.0 cm, wide diameter to the soil 21.5 cm) were installed on the forest surface. The collection was performed from May 5 to August 7 for 25 years, from 1986 to 2010. The distances between traps were about 10 m. The traps were set at the same locations every year to ensure continuity for the census data, and samples were collected every second day. The collected specimens were preserved in sample bottles with 70% ethanol and identified to the level of species under a stereomicroscope in the laboratory. Their sex was identified based on morphology.

The study site was located in a west-facing pine forest at an elevation of 78–98 m. The understory of the forest consisted of small trees and shrubs, such as *Robinia pseudoacacia* L*., Lespedeza bicolour* Turcz*.*, and *Rubus idaeus* L*.,* which together accounted for less than 50% of the ground coverage. No biological control using parasitoids was conducted to control the PNGM in the forest during the monitoring period.

Although the invasive history of the study site has not been comprehensively reported, those of other local areas have been reported in detail (Lee et al., 1997). The first occurrence of PNGM in the study area was inferred in 1975. Therefore, the monitoring for this study was begun at 10 years, at the most, of the initial invasion, and the PNGM density was in the decreasing phase, considering that the outbreak of PNGM generally occurs within six or seven years after invasion of a new area (Park et al., 2001).

### Environmental factors

To evaluate the influence of environmental factors on the structure and dynamics of the community, we chose four meteorological factors on the basis of PNGM biology: mean of daily minimum temperature in January (minimum temperature), mean of daily maximum temperature in July and August (maximum temperature), annual mean temperature (mean temperature), and precipitation in spring from March to May (precipitation). The minimum, maximum, and mean temperatures and the precipitation affect mortality of PNGM in winter, the mortality of egg and young larvae of PNGM, and the phenology and emergence of PNGM, respectively. The meteorological data were obtained from the Korean Meteorological Administration (KMA; http://www.kma.go.kr) measured at the Yeongcheon (35°58′N, 128°57′E) weather station, located approximately 12 km from the study site.

### Data analysis

The parasitism rate of each parasitoid was estimated based on the number of adult parasitoids and the number of adult PNGMs collected in the monitoring traps, as follows: *P*_*i*_ = *n*_*i*_∕(*N*_*p*_ + *N*_*h*_), where *P*_*i*_ is the parasitism rate of the species *i*, *n*_*i*_ is the number of parasitoid species *i,* and *N*_*p*_ and *N*_*h*_ are the number of parasitoids and host (PNGM) per trap for each year, respectively. The samples from all the 20 traps in each year were pooled because of the limited experimental feasibility for handling all the samples separately, and the mean abundance of the parasitoids and host (per trap) were used for the calculation of parasitism rate.

Typically, *I. seoulis* is a solitary parasitoid, whereas *P. matsutama* is generally gregarious (Jeon et al., 1985). To avoid overestimation of the rate of parasitism by *P. matsutama* due to superparasitism, the number of parasitoids collected in traps was divided by 1.32, which is the average number of parasitoids that emerge from one host (Son, Chung & Lee, 2012). The rate of change in the PNGM population was estimated by dividing the PNGM abundance in the year *t* with the PNGM abundance in the year *t* − 1.

The relationships between the PNGM abundance and parasitism rates, and the numerical response of parasitoids to host abundance were analysed with linear regression analyses (Soné, 1986) using SAS statistical software, and the data were arcsine root transformed before the statistical analysis. The relationships between the Julian date and the emergence periods of PNGM and the three parasitoids were analysed by a cumulative Weibull function as shown in Eq. (1):

(1) \begin{eqnarray*}C=1-\mathrm{exp}[-{\{(JD-r)/c\}}^{b}]\end{eqnarray*}

where *C* is the cumulative proportion of the PNGM or its adult parasitoids at a given Julian date (*JD*), *r* is the expected Julian date at the onset of first emergence, *c* is the constant for rate of emergence, and *b* is the parameter for shape. The equation was fitted by the Marquardt method using the SAS program.

To evaluate the relationships between PNGM, its three parasitoids, and the environmental factors, principal component analysis (PCA) was conducted with the abundance data of PNGM and its parasitoids. The abundance data were rescaled for a trap for each year. After the PCA ordination, a biplot with the meteorological factors was presented on the PCA by calculating the correlation coefficients between the first two PCA axes and meteorological factors, to characterize their relationships. The abundance data showing high variations were transformed based on the natural logarithm prior to the PCA. Before the log-transformation, the number 1 was added to the abundance values to avoid the logarithm of zero. The randomization test was used to evaluate whether PCA extracted stronger axes than expected by chance, with 999 iterations. The PCA was conducted using PC-ORD software version 5.31.

## Results

**Figure 1:**
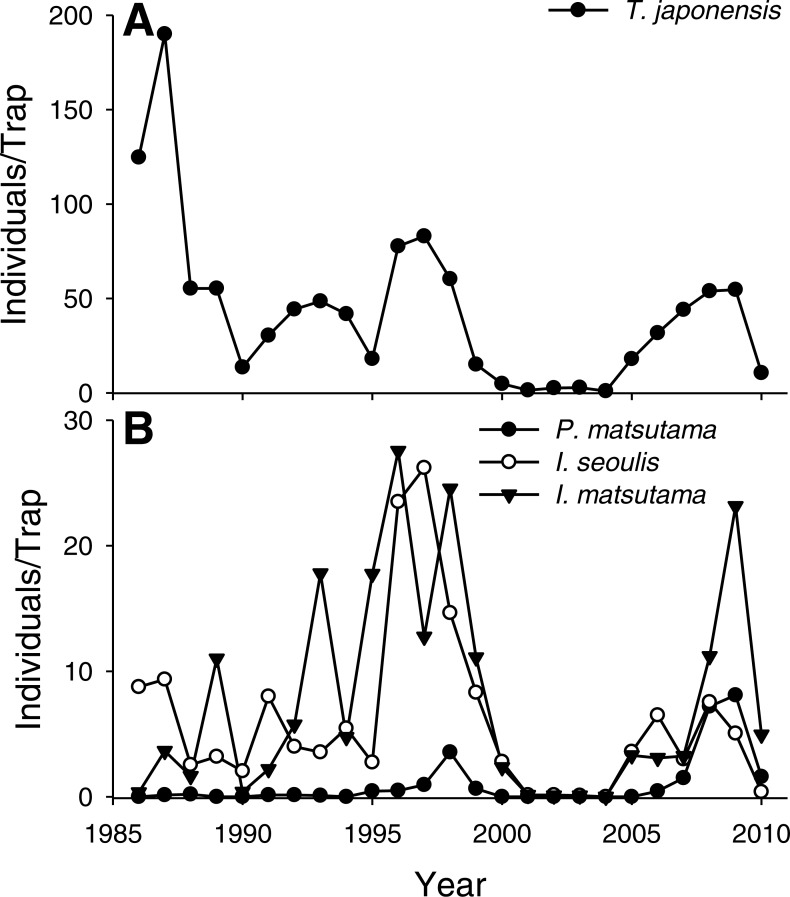
Changes in the abundance of pine needle gall midge (PNGM) (A) and its parasitoids (B), in the study site from 1986 to 2010. The samples from all the traps were pooled each year, and the abundance of each species was divided by the number of traps used. Therefore, the variance for each abundance is not provided.

**Figure 2:**
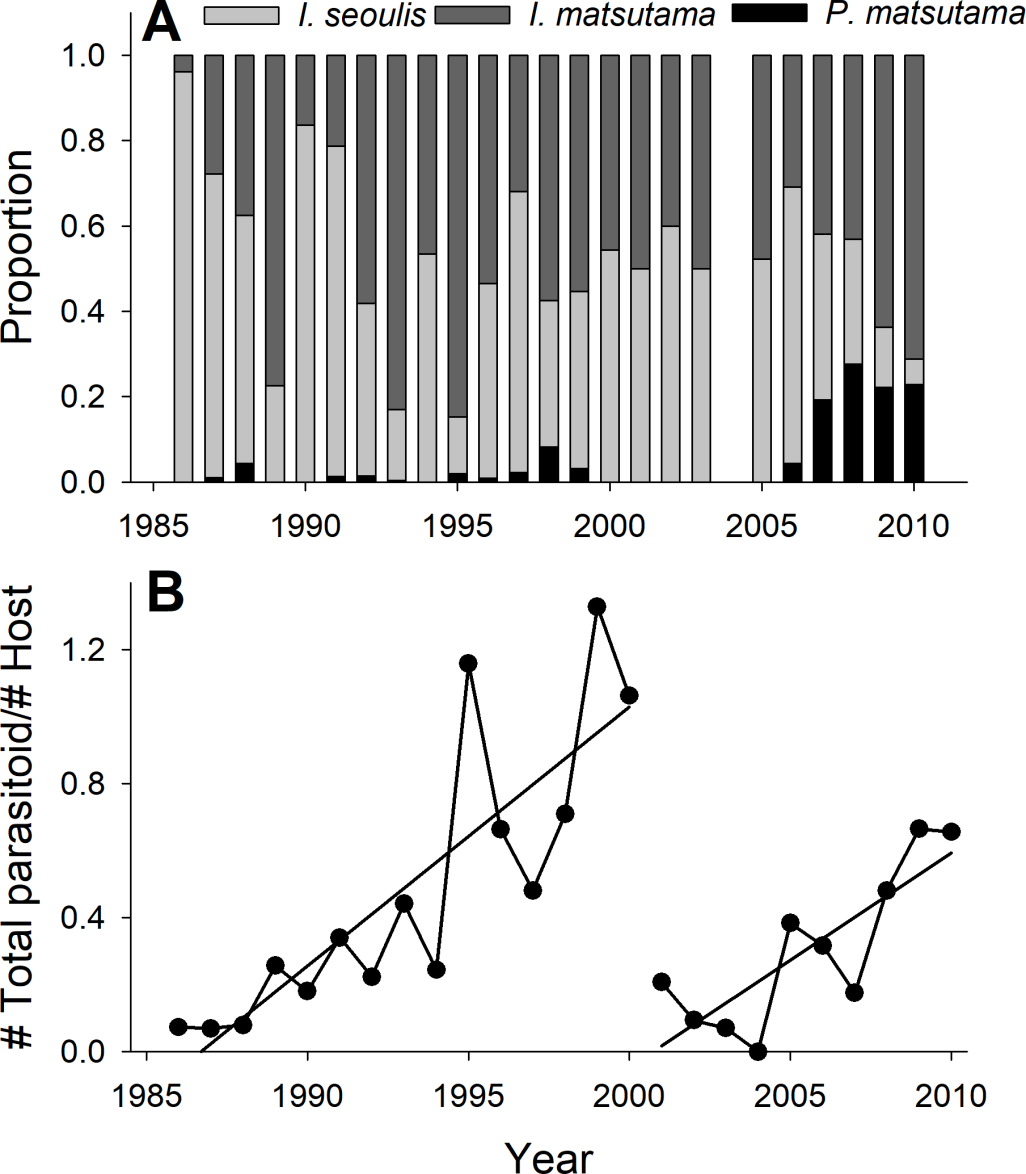
Changes in the relative abundance of each parasitoid (A), and host-parasitoid ratio (B).

**Figure 3:**
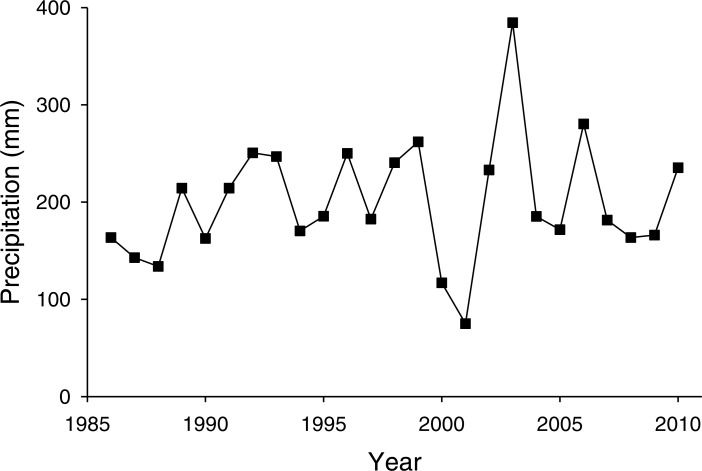
Changes in precipitation during spring (March–May) measured at Yeongcheon weather station near the study site over the study period (1986–2010). Data were obtained from the Korean Meteorological Administration (KMA; http://www.kma.go.kr).

The PNGM density was high in 1986 and 1987, with more than 120 individuals/trap/year; thereafter, the density fluctuated with approximately 33.5 individuals/trap/year. The density of *I. matsutama* and *I. seoulis* remained around nine individuals/trap/year, whereas that *P. matsutama* was relatively lower, and before 2004, it was only observed occasionally. The PNGM density decreased from 2000 to 2002, resulting in the collapse of its parasitoid community. Therefore, the density of parasitoids as well as the proportion of *P. matsutama* increased (Figs. 1 and 2, Table S1). The parasitoid:host ratio increased linearly from 1986 to 1999; it then decreased dramatically from 2001 to 2004, and then increased linearly from 2005 onward (Fig. 2B). The precipitation conspicuously decreased in the spring of 2000 and 2001 (Fig. 3, Table S2).

**Figure 4:**
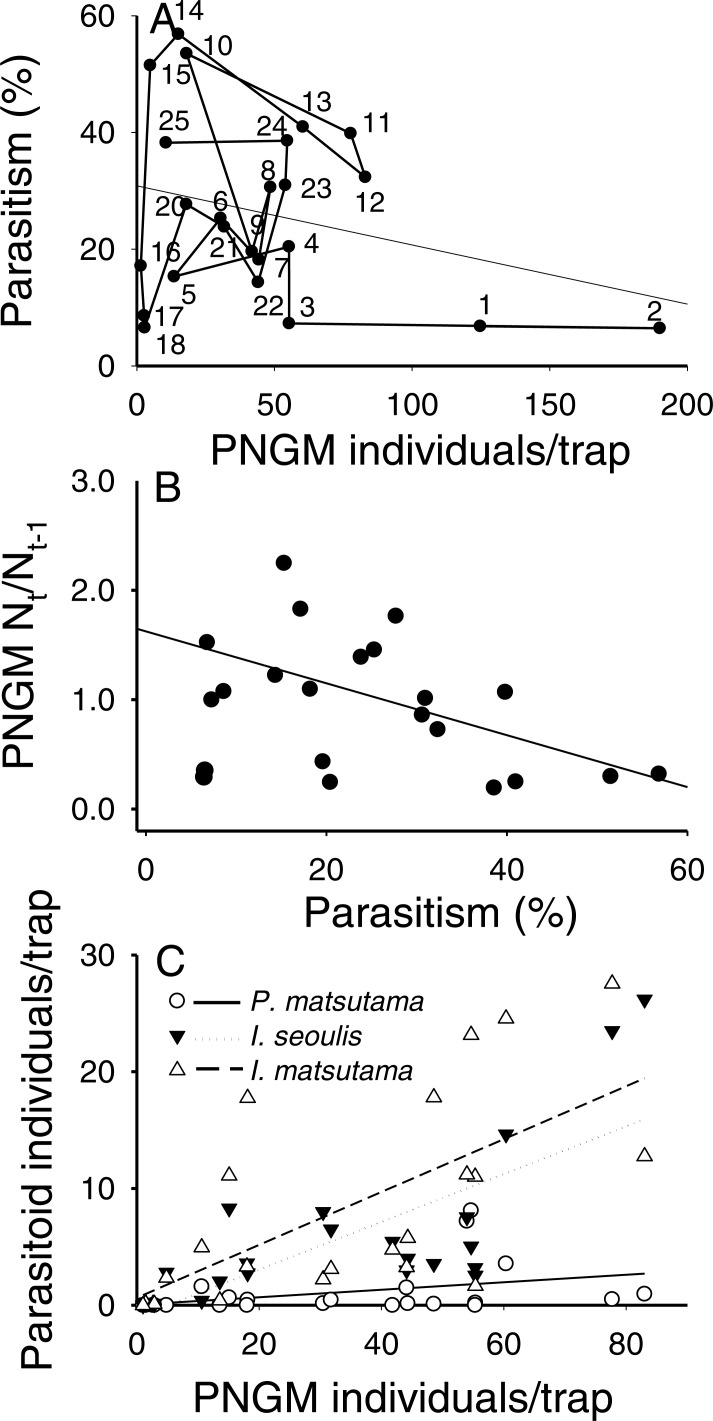
Relationship between the pine needle gall midge (PNGM) abundance and total parasitism rates (*r*^2^ = 0.26; *F*_1,18_ = 6.31; *P* < 0.021) (A), influence of total parasitism rate on the changes in PNGM (*r*^2^ = 0.28; *F*_1,18_ = 8.31; *P* < 0.01) (B), and numerical response of parasitoids to host abundance (*I. matsutama*: *r*^2^ = 0.44, *F*_1,21_ = 16.50, *P* < 0.0001; *I. seoulis*: *r*^2^ = 0.56, *F*_1,21_ = 26.38, *P* < 0.0001; *P. matsutama*: *r*^2^ = 0.13, *F*_1,21_ = 3.24, *P* = 0.08) (C). The numbers in (A) indicate the years from the first survey (eg., 1, 1986; 2, 1997; 25, 2010).

The total parasitism rate for the three parasitoids decreased linearly as a function of PNGM abundance (*r*^2^ = 0.26; *F*_1,18_ = 6.31; *P* < 0.021), demonstrating a delayed, inverse density-dependence and with no clear equilibrium in yearly change (Fig. 4A). As the total parasitism rate increased, the rate of change in the PNGM density decreased linearly (*r*^2^ = 0.28; *F*_1,18_ = 8.31; *P* < 0.01) (Fig. 4B). Meanwhile, the three parasitoid species displayed different responses to their host density. The numerical responses of *I. matsutama* and *I. seoulis* increased linearly as functions of PNGM abundance with similar increasing rate (*I. matsutama*: *r*^2^ = 0.44; *F*_1,21_ = 16.50; *P* < 0.0001; *I. seoulis*: *r*^2^ = 0.56; *F*_1,21_ = 26.38; *P* < 0.0001), whereas that of *P. matsutama* was not statistically significant with relatively low increasing rate (*r*^2^ = 0.13; *F*_1,21_ = 3.24; *P* = 0.08) (Fig. 4C).

**Figure 5:**
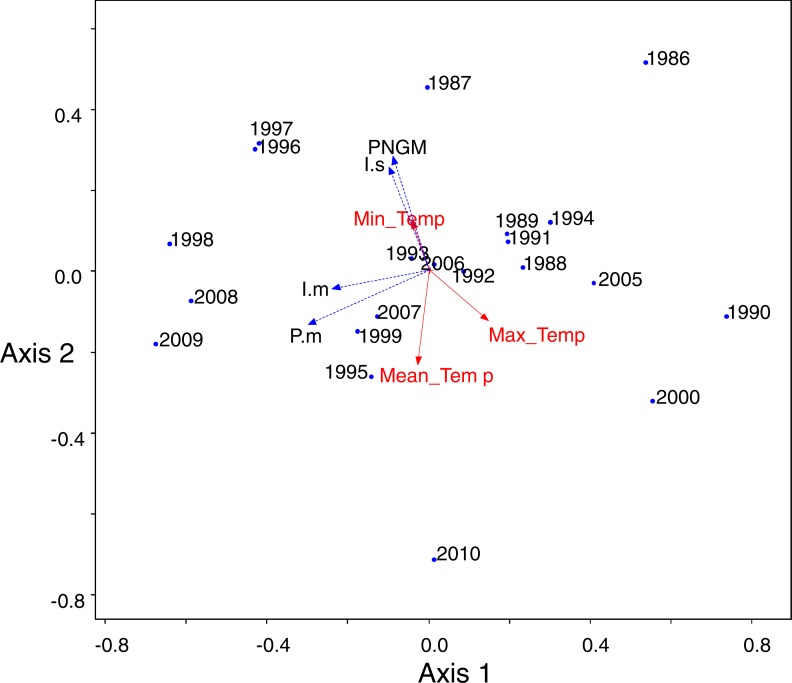
Principal component analysis (PCA) ordination of the sampling years based on the differences of community consisting of three parasitoids and their host. Meteorological factors were biploted in the PCA through correlation coefficients between coordinates of each axis (1 and 2) and each meteorological factor in each year. The eigenvalues for axes 1 and 2 were 2.00 (49.9% of variance, *P* = 0.006 by randomization test) and 1.20 (29.93%, *P* = 0.216), respectively. Blue and red colors represented biological and abiotic factors, respectively. PNGM, pine needle gall midge (host); *P.m.*, *Platygaster matsutama*; *I.s.*, *Inostemma seoulis*; and *I.m.*, *Inostemma matsutama*. Min_Temp, mean of daily minimum temperature in January; Max_Temp, mean of daily maximum temperature; and Mean_Temp, annual mean temperature.

PCA was conducted to characterize the temporal changes in the community (Fig. 5). The data from 2001 to 2004 were excluded from the analysis because of the collapse of populations. The yearly changes in the community were reflected in the ordination (eigenvalues: axis 1: 3.18 (54.8% of variance, *P* = 0.012 by randomization test) and axis 2: 1.47 (25.3%, *P* = 0.808)). The samples with low density of parasitoids and their host were assigned to the lower right, whereas the samples with high density were to the upper left. The density of *I*. *seoulis* was strongly associated with the abundance of PNGM because they located close each other (*r* = 0.53, *p* = 0.01), whereas *I. matsutama* and *P. matsutama* had relatively low association on the abundance of PNGM (*r* = 0.21, *P* = 0.30 and *r* = 0.10, *P* = 0.64, respectively). However, these two parasitoids strongly associated with each other (*r* = 0.50, *P* = 0.01). Based on the PCA ordination, the abundance of PNGM and *I. seoulis* negatively associated with the mean temperature (Fig. 5), although their correlation coefficients were not statistically significant (*r* =  − 0.38, *P* = 0.06 and *r* =  − 0.13, *P* = 0.53).

**Figure 6:**
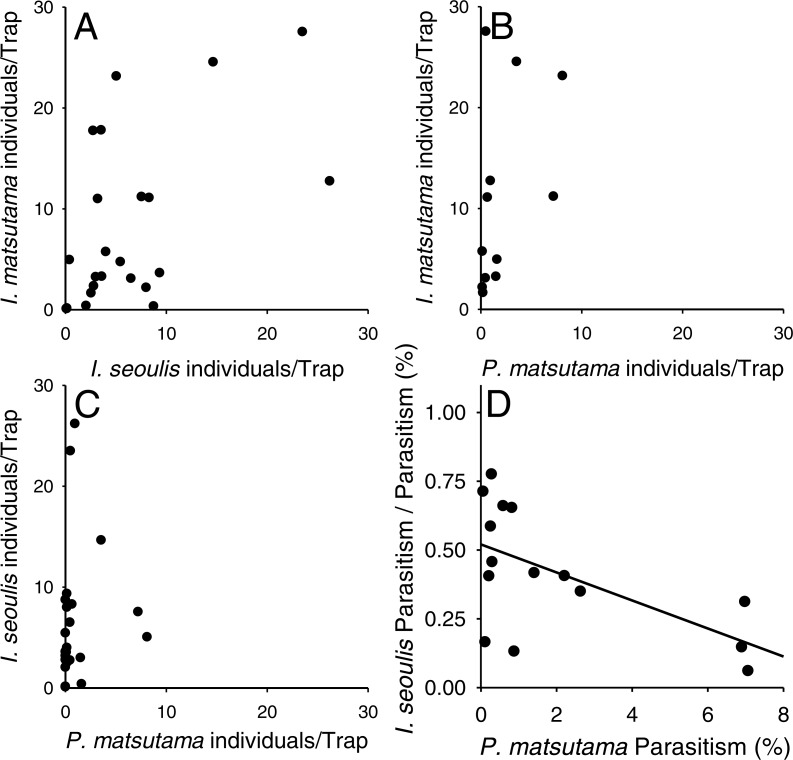
Relationship between two parasitoids: *Inostemma matsutama* and *Platygaster matsutama* (A), *I. matsutama* and *Inostemma seoulis* (B), *I. matsutama* and *P. matsutama* (C), and relationship between the proportion of *I. seoulis* in total parasitism and *P. matsutama* parasitism rate (D).

**Figure 7:**
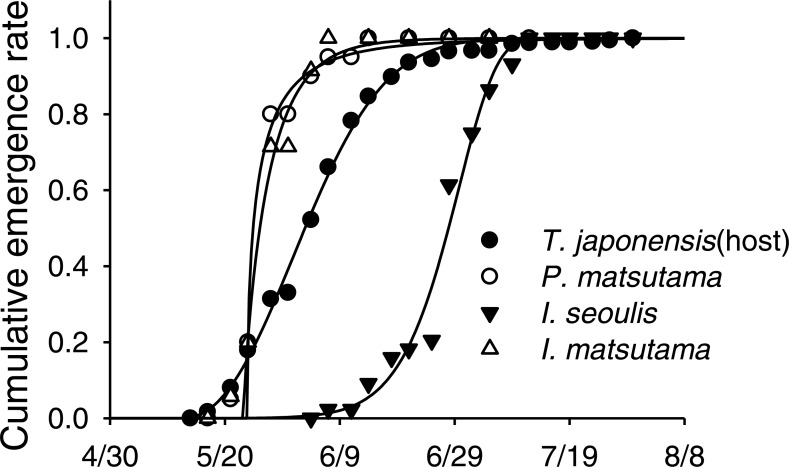
An example of emergence patterns for pine needle gall midge (PNGM) and its parasitoids in 2007.

Neither of the two dominant parasitoids, *I. matsutama* nor *I. seoulis*, was a superior competitor based on their densities (Fig. 6A). In contrast, both *I. matsutama* (which emerges during a similar period) and *I. seoulis* were superior competitors to *P. matsutama* (Figs. 6B and 6C). Interestingly, the increasing rate of parasitism by *P. matsutama* correlated with a decreasing rate of parasitism by *I. seoulis* in the total parasitism rate (*r*^2^ = 0.36; *F*_1,13_ = 7.21; *P* < 0.019) (Fig. 6D). The emergence curves of PNGM and its parasitoids were well fitted by the cumulative Weibull function (*r*^2^ > 0.98). Emergence periods of *I. matsutama* and *P. matsutama* were similar to and earlier than that of PNGM, respectively, whereas the emergence period of *I. seoulis* was later than that of PNGM (Fig. 7).

## Discussion

The structural dynamics of PNGM and its parasitoid community was influenced by the biological traits of the three parasitoids during the invasion of PNGM, and was mediated by the environmental factors. Following the outbreak of PNGM, differences in the establishment time and phenology of the parasitoids, which are associated with interspecific competition patterns, determined their community composition and dynamics before the collapse of the PNGM population and that of its parasitoids during the drought from 2000 to 2002. After the drought, the composition of the parasitoids was changed, indicating that *P. matsutama* and *I. matsutama* were superior to *I. seoulis* in the exploitative competition.

### Host–parasitoid interactions and invasion history

Both PNGM and its parasitoid community displayed cyclic oscillations in abundance (Fig. 1). Interestingly, the host–parasitoid ratio increased from 1986 and 1987 (the years of PNGM outbreak) until 1999 (Fig. 2B); thereafter the ratio was dramatically decreased until 2004, after which it increased again. The initial increase in the ratio was due to the invasion, whereas the second increase was probably due to the external factors, such as environmental stress, including drought.

**Figure 8:**
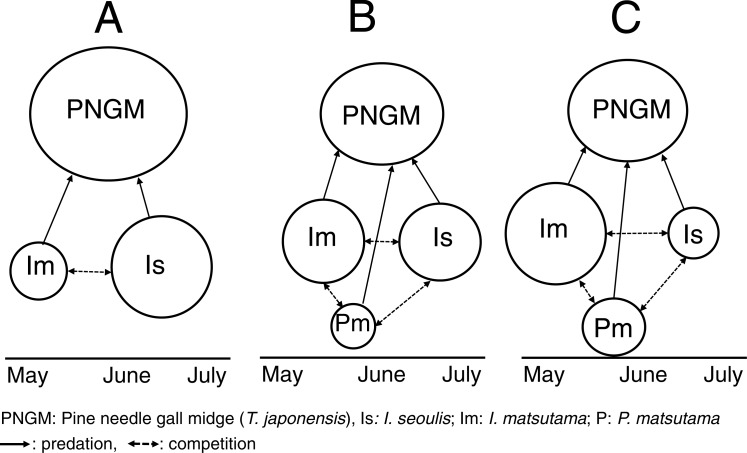
Schematic diagram for changes in the community structure of parasitoids on pine needle gall midge (PNGM) with time. (A) Initial stage of PNGM invasion, (B) intermediate stage, and (C) most recent stage. Different sizes of symbols indicate relative population sizes.

Based on our results, we propose that the invasion history of PNGM determined the community structure of the parasitoids (Fig. 8). During the early stage of PNGM invasion, the difference in the establishment time of the community among the three parasitoids might influence the community structure. *I. seoulis* was one of the the three parasitoids established on PNGM; the geographical distribution of *I. seoulis* overlaped with that of PNGM in 1985, whereas *I. matsutama* and *P. matsutama* were restricted to southern Korea (Table 1). The PNGM invaded Korea in 1929 and spread more than 80% of the pine forests of South Korea in 1984 (Choi & Park, 2012). While the parasitism by *I. seoulis* during the early stage of PNGM invasion was relatively high (Fig. 1), the parasitism by *I. matsutama* and *P. matsutama* increased several years later, indicating that these parasitoids began to attack this host later (Fig. 1). The parasitism by *I. seoulis* has been reported to be higher than that by the other parasitoids in areas where PNGM had invaded recently (Park et al., 2001). This early establishment of *I. seoulis* might be the main reason for its initial dominance during the PNGM invasion.

In this study, the parasitism rates for all the three species were inversely related with the PNGM abundance; high PNGM abundance was observed during the initial stage of PNGM invasion, but the abundance decreased with the increasing parasitism, suggesting that the PNGM abundance is regulated by its parasitoids (Choi et al., 2011). The parasitism rates for *I. seoulis* and *I. matsutama* increased in the increase in total parasitism (Fig. 3C), indicating that the influence of competition between the parasitoids on the total parasitism rate was not significant (Jeon et al., 2006; Soné, 1986). Our results suggest that intense interspecific competition between *I. matsutama* and *I. seoulis* might have been mitigated by niche partitioning, contributing to a more efficient suppression of the PNGM abundance than would be achieved by one species.

**Table 1:** Life history traits of *Inostemma seoulis*, *Inostemma matsutama*, and *Platygaster matsutama* based on the literature.

Characteristic	Parasitoid species			Reference
	*Platygaster matsutama*	*Inostemma seoulis*	*Inostemma matsutama*	
Lower developmental threshold temperature (LDT; °C)	4.2	8.4	Unknown	Son, Chung & Lee (2012)
Degree-days to complete development (DD)	741.2	946.1	Unknown	Son, Chung & Lee (2012)
Optimum temperature (° C)	24.8	26.5	Unknown	Son, Chung & Lee (2012)
Number of progeny/host	Up to 4	1	Unknown	Jeon et al. (1985)
Attack stage	Egg	Egg and larva	1st instar larva	Jeon et al. (1985)
Searching efficiency	Low	High	Unknown	Jeon et al. (1985)
Sex ratio (Female: Male)	38:62	60:40	Unknown	Jeon et al. (1985)
Egg production	Pro-ovigeny	Pro-ovigeny	Synovigeny	Jeon et al. (1985) and FES (1975)
Maximum number of ovarian eggs	1,569	555	Unknown	Jeon et al. (1985)
Number of ovarian eggs	1,078–2,374	293–772	Unknown	Jeon et al. (1985)
Observed parasitoid/host ratio	1.32:1	1:1	Unknown	Jeon et al. (1985)
Observed percentage of superparasitism	32.1–65.5	None	Unknown	Jeon et al. (1985)
Year of first report	1979	1962	1979	Jeon et al. (1985)
Overwintering stage	Embryo	Larva	Larva	Jeon et al. (1985)
Distribution^a^	Southern Korea	National-wide	Southern Korea	Jeon et al. (1985)

**Notes.**

a Distribution of three parasitoids based on Jeon et al. (1985).

Although the origins of PNGM and its parasitoids are unclear, based on the synchronous phenology of these species, it is suspected that they were from Japan. The parasitoids of PNGM lay their eggs in the eggs or first-instar larvae of PNGM. When the larvae of PNGM reach maturity, the parasitoid eggs hatch and the the parasitoids overwinter in the host body (Jeon et al., 1985). Such synchrony suggests that the three parasitoids coevolved and invaded the new habitat with their host. A similar instance can be found with another platygastrid parasitoid*, Platygaster robiniae* Buhl & Duso*,* which synchronized with and invaded Italy and Korea with its host, *Obolodiposis robiniae* (Haldeman) (Kim et al., 2012; Buhl & Duso, 2008).

### Influence of environmental factors

A severe drought induced the collapse of the community from 2000 to 2004, and influenced the parasitoid community. Severe spring droughts were reported in southern Korea from 2000 to 2002, with the drought in 2001 being the worst in Korea in last 90 years (Kim et al., 2011). Because high content of soil moisture provides favourable condition for overwintering PNGM (Chung & Hyun, 1986), the drought of three years disturbed the populations of both the PNGM and its parasitoids, and the community could only recover in 2005, probably due to the delayed effects. The changes in the parasitoid community structure could also be attributed to the meteorological factors. In the PCA ordination, the abundance of PNGM and *I. seoulis* associated negatively with the mean temperature (Fig. 5), suggesting that the fitness of each parasitoid could be dependent on the temperature.

The parasitism rate of *P. matsutama* was higher in southern Korea in 1977, suggesting that the field performance of this species is better in warmer areas (FES, 1975). On the contrary, the lower threshold temperature for the development of *P. matsutama* was lower than that of *I. seoulis* (Jeon, 1991) (Table 1). The different responses of parasitoids to temperature could reflect phenology rather than the temperature tolerance, as the emergence period of *P. matsutama* was earlier than that of *I. seoulis.* In addition, high temperature in late June and July might have led to a decrease in parasitism by *I. seoulis.* The development of *I. seoulis* and *P. matsutama* ceases at 30 °C, and the inhibition of development in *I. seoulis* and *P. matsutama* occurs only at 26.6 and 24.9 °C, respectively (Jeon, 1991). It is possible that *P. matsutama* simply avoids high temperature because it emerges 2–4 weeks earlier than *I. seoulis.* The results suggest that PNGM and *I. seoulis* are that are adapted to cool, while *P. matsutama* and *I. matsutama* performed better under warmer condition. Considering the increase in mean temperature (0.85 °C) in the study area, over the last 25 years, this has the potential of conferring a fitness advantage on the heat-tolerant species in the community, following a community collapse.

### Influence of competition on the host–parasitoid interactions

The differences in the competitive superiority of the parasitoids determined the parasitoid community structure after the establishment of *I. seoulis.* After the PNGM outbreak, *I. matsutama* and *I. seoulis* were dominant because they were equivalent competitors, whereas *P. matsutama* was a relatively weak competitor. After the complete collapse of the parasitoid community in 2004, the parasitism rate for *P. matsutama* increased and that for *I. seoulis* decreased. The emergence period of *P. matsutama* was similar to that of *I. matsutama*, but earlier than that of *I. seoulis*, suggesting that the early-emergence of *I. matsutama* and *P. matsutama* limited the availability of PNGM to the relatively late-emerging *I. seoulis*. Thus, the increased parasitism rate by *P. matsutama* induced a decrease in the dominance by *I. seoulis*.

In our study, the species with the most restricted distribution, *P. matsutama*, likely suffered from intense interspecific competition with *I. matsutama* because of the overlap of their phenology. Throughout the 25 years of this study, *P. matsutama* has only been periodically observed, and this pattern might have occurred because of two reasons: either the *P. matsutama* population was excluded by *I. matsutama* and was periodically introduced from outside of the site or it existed in the forest at a low abundance because it is specialist parasitoid of PNGM (Yoshida & Hirashima, 1979). The divergence of life history between the parasitoids suggests that the *P. matsutama* population existed at a low abundance and was undetected during trapping over several years.

During the invasion, the structural dynamics of PNGM and its parasitoid community was influenced by differences in the biological traits of parasitoids: the timing for the initial establishment of the host–parasitoid association was a major factor in the initial stage of a PNGM invasion; thereafter, incomplete superiority in competition was a determining factor; following collapse due to a severe drought, indirect competition by a combination of parasitoids determined the community structure. This pattern shows that the history of community change is explained by the diverse traits of its community members, as mediated by environmental factors, such as drought and temperature.

##  Supplemental Information

10.7717/peerj.3610/supp-1 Table S1 Raw data: community data Changes in abundance (individuals/trap) of host (*Thecodiplosis japonensis*) and its three parasitoids in the study site from 1986 to 2010.

10.7717/peerj.3610/supp-2 Table S2 Raw data: meteorological data Changes of meteorological factors measured at Yeongcheon weather station near the study site during the study period 1986–2010. Data were obtained from the Korean Meteorological Administration (KMA; http://www.kma.go.kr). Minimum temperature: mean of daily minimum temperature in January, maximum temperature: mean of daily maximum temperature in July and August, mean temperature: annual mean temperature, and precipitation: precipitation in spring from March to May.

## Additional Information and Declarations

### Data Availability

The following information was supplied regarding data availability: The raw data has been supplied as Supplementary Files.
