# Supplementary material for: Structural dynamics in the host-parasitoid system of the pine needle gall midge (Thecodiplosis japonensis) during invasion
Source: PeerJ. 2017 Aug 22;5:e3610. doi: 10.7717/peerj.3610 (PMC5571814; doi:10.7717/peerj.3610)
Supplement: Table S1 — Changes in abundance (individuals/trap) of host (Thecodiplosis japonensis) and its three parasitoids in the study site from 1986 to 2010. [file peerj-05-3610-s001.docx]

Table S1. Changes in abundance of host (*Thecodiplosis japonensis*) and its three parasitoids collected with 20 traps in the study site. The collection was performed from May 5 to August 7 for 25 years, from 1986 to 2010.

| Year | Month | Day | *Thecodiplosis japonensis* | | *Platygaster matsutama* | | *Inostemma seoulis* | | *Inostemma matsutama* | |
| --- | --- | --- | --- | --- | --- | --- | --- | --- | --- | --- |
|  |  |  | Female | Male | Female | Male | Female | Male | Female | Male |
| 1986 | 5 | 18 | 1 | 1 | 0 | 0 | 0 | 0 | 0 | 0 |
| 1986 | 5 | 20 | 2 | 3 | 0 | 0 | 0 | 0 | 0 | 0 |
| 1986 | 5 | 22 | 23 | 12 | 0 | 0 | 0 | 0 | 0 | 0 |
| 1986 | 5 | 23 | 131 | 90 | 0 | 0 | 0 | 0 | 0 | 0 |
| 1986 | 5 | 24 | 127 | 116 | 0 | 0 | 0 | 0 | 0 | 0 |
| 1986 | 5 | 26 | 296 | 131 | 0 | 0 | 0 | 0 | 0 | 0 |
| 1986 | 5 | 28 | 142 | 81 | 0 | 0 | 0 | 0 | 1 | 0 |
| 1986 | 5 | 30 | 59 | 38 | 0 | 0 | 0 | 0 | 1 | 0 |
| 1986 | 6 | 1 | 298 | 147 | 0 | 0 | 0 | 0 | 1 | 0 |
| 1986 | 6 | 3 | 104 | 59 | 0 | 0 | 0 | 0 | 0 | 1 |
| 1986 | 6 | 4 | 28 | 38 | 0 | 0 | 0 | 0 | 0 | 0 |
| 1986 | 6 | 5 | 51 | 30 | 0 | 0 | 0 | 0 | 1 | 0 |
| 1986 | 6 | 7 | 6 | 16 | 0 | 0 | 0 | 0 | 2 | 0 |
| 1986 | 6 | 9 | 87 | 57 | 0 | 0 | 1 | 1 | 0 | 0 |
| 1986 | 6 | 11 | 59 | 47 | 0 | 0 | 2 | 3 | 0 | 0 |
| 1986 | 6 | 13 | 28 | 34 | 0 | 0 | 6 | 1 | 0 | 0 |
| 1986 | 6 | 18 | 30 | 19 | 0 | 0 | 26 | 11 | 0 | 0 |
| 1986 | 6 | 20 | 20 | 16 | 0 | 0 | 10 | 9 | 0 | 0 |
| 1986 | 6 | 26 | 13 | 4 | 0 | 0 | 11 | 7 | 0 | 0 |
| 1986 | 6 | 27 | 5 | 2 | 0 | 0 | 5 | 5 | 0 | 0 |
| 1986 | 6 | 28 | 0 | 0 | 0 | 0 | 1 | 0 | 0 | 0 |
| 1986 | 6 | 30 | 3 | 2 | 0 | 0 | 17 | 8 | 0 | 0 |
| 1986 | 7 | 2 | 4 | 3 | 0 | 0 | 13 | 6 | 0 | 0 |
| 1986 | 7 | 4 | 4 | 5 | 0 | 0 | 10 | 6 | 0 | 0 |
| 1986 | 7 | 7 | 5 | 3 | 0 | 0 | 5 | 1 | 0 | 0 |
| 1986 | 7 | 9 | 2 | 2 | 0 | 0 | 4 | 0 | 0 | 0 |
| 1986 | 7 | 11 | 4 | 0 | 0 | 0 | 3 | 0 | 0 | 0 |
| 1986 | 7 | 14 | 1 | 1 | 0 | 0 | 1 | 0 | 0 | 0 |
| 1986 | 7 | 16 | 1 | 0 | 0 | 0 | 0 | 0 | 0 | 0 |
| 1986 | 7 | 18 | 0 | 0 | 0 | 0 | 0 | 0 | 0 | 0 |
| 1986 | 7 | 21 | 1 | 0 | 0 | 0 | 1 | 0 | 0 | 0 |
| 1986 | 7 | 23 | 1 | 0 | 0 | 0 | 1 | 0 | 0 | 0 |
| 1986 | 7 | 25 | 1 | 0 | 0 | 0 | 0 | 0 | 0 | 0 |
| 1986 | 7 | 28 | 0 | 0 | 0 | 0 | 0 | 0 | 0 | 0 |
| 1986 | 7 | 30 | 0 | 1 | 0 | 0 | 0 | 0 | 0 | 0 |
| 1986 | 8 | 1 | 0 | 0 | 0 | 0 | 0 | 0 | 0 | 0 |
| 1987 | 5 | 8 | 0 | 0 | 0 | 1 | 0 | 0 | 0 | 0 |
| 1987 | 5 | 12 | 0 | 0 | 0 | 0 | 0 | 0 | 0 | 0 |
| 1987 | 5 | 14 | 0 | 0 | 0 | 0 | 0 | 0 | 0 | 0 |
| 1987 | 5 | 15 | 0 | 0 | 0 | 0 | 0 | 0 | 0 | 0 |
| 1987 | 5 | 16 | 0 | 0 | 0 | 0 | 0 | 0 | 0 | 0 |
| 1987 | 5 | 17 | 5 | 3 | 0 | 0 | 0 | 0 | 0 | 0 |
| 1987 | 5 | 19 | 13 | 10 | 0 | 1 | 0 | 0 | 0 | 0 |
| 1987 | 5 | 20 | 35 | 21 | 0 | 0 | 0 | 0 | 0 | 0 |
| 1987 | 5 | 21 | 47 | 24 | 0 | 1 | 0 | 0 | 1 | 3 |
| 1987 | 5 | 23 | 200 | 80 | 0 | 0 | 0 | 0 | 0 | 5 |
| 1987 | 5 | 25 | 238 | 90 | 0 | 0 | 0 | 0 | 2 | 5 |
| 1987 | 5 | 27 | 272 | 190 | 0 | 0 | 0 | 0 | 3 | 14 |
| 1987 | 5 | 28 | 359 | 185 | 0 | 0 | 0 | 0 | 9 | 14 |
| 1987 | 5 | 29 | 92 | 61 | 0 | 0 | 0 | 0 | 6 | 3 |
| 1987 | 5 | 31 | 171 | 87 | 0 | 0 | 0 | 0 | 2 | 0 |
| 1987 | 6 | 1 | 200 | 150 | 0 | 0 | 0 | 0 | 2 | 1 |
| 1987 | 6 | 2 | 83 | 50 | 0 | 0 | 0 | 0 | 1 | 1 |
| 1987 | 6 | 3 | 22 | 20 | 0 | 0 | 0 | 0 | 1 | 0 |
| 1987 | 6 | 4 | 156 | 153 | 0 | 0 | 0 | 0 | 0 | 0 |
| 1987 | 6 | 5 | 62 | 60 | 0 | 0 | 0 | 0 | 0 | 0 |
| 1987 | 6 | 8 | 14 | 9 | 0 | 0 | 0 | 0 | 0 | 0 |
| 1987 | 6 | 9 | 18 | 8 | 0 | 0 | 0 | 0 | 0 | 0 |
| 1987 | 6 | 10 | 37 | 20 | 0 | 0 | 0 | 1 | 0 | 0 |
| 1987 | 6 | 11 | 42 | 31 | 0 | 0 | 1 | 4 | 0 | 0 |
| 1987 | 6 | 12 | 28 | 10 | 0 | 0 | 0 | 0 | 0 | 0 |
| 1987 | 6 | 14 | 37 | 21 | 0 | 0 | 7 | 3 | 0 | 0 |
| 1987 | 6 | 15 | 6 | 5 | 0 | 0 | 3 | 3 | 0 | 0 |
| 1987 | 6 | 16 | 15 | 7 | 0 | 0 | 2 | 0 | 0 | 0 |
| 1987 | 6 | 17 | 21 | 13 | 0 | 0 | 1 | 2 | 0 | 0 |
| 1987 | 6 | 18 | 20 | 6 | 0 | 0 | 3 | 1 | 0 | 0 |
| 1987 | 6 | 19 | 10 | 4 | 0 | 0 | 4 | 4 | 0 | 0 |
| 1987 | 6 | 22 | 31 | 31 | 0 | 0 | 6 | 3 | 0 | 0 |
| 1987 | 6 | 24 | 39 | 18 | 0 | 0 | 9 | 8 | 0 | 0 |
| 1987 | 6 | 26 | 25 | 12 | 0 | 0 | 17 | 14 | 0 | 0 |
| 1987 | 6 | 27 | 9 | 8 | 0 | 0 | 17 | 10 | 0 | 0 |
| 1987 | 6 | 29 | 19 | 4 | 0 | 0 | 10 | 6 | 0 | 0 |
| 1987 | 7 | 1 | 9 | 5 | 0 | 0 | 18 | 8 | 0 | 0 |
| 1987 | 7 | 3 | 8 | 1 | 0 | 0 | 5 | 2 | 0 | 0 |
| 1987 | 7 | 4 | 11 | 3 | 0 | 0 | 1 | 3 | 0 | 0 |
| 1987 | 7 | 6 | 12 | 4 | 0 | 0 | 0 | 0 | 0 | 0 |
| 1987 | 7 | 8 | 9 | 1 | 0 | 0 | 7 | 0 | 0 | 0 |
| 1987 | 7 | 10 | 7 | 3 | 0 | 0 | 2 | 0 | 0 | 0 |
| 1987 | 7 | 13 | 2 | 0 | 0 | 0 | 0 | 0 | 0 | 0 |
| 1987 | 7 | 15 | 4 | 0 | 0 | 0 | 0 | 0 | 0 | 0 |
| 1987 | 7 | 18 | 2 | 0 | 0 | 0 | 1 | 0 | 0 | 0 |
| 1987 | 7 | 20 | 0 | 1 | 0 | 0 | 0 | 0 | 0 | 0 |
| 1987 | 7 | 27 | 0 | 1 | 0 | 0 | 0 | 0 | 0 | 0 |
| 1987 | 7 | 29 | 0 | 0 | 0 | 0 | 0 | 1 | 0 | 0 |
| 1988 | 5 | 15 | 1 | 0 | 0 | 0 | 0 | 0 | 0 | 0 |
| 1988 | 5 | 16 | 0 | 2 | 3 | 0 | 0 | 0 | 0 | 0 |
| 1988 | 5 | 17 | 2 | 4 | 0 | 0 | 0 | 0 | 0 | 0 |
| 1988 | 5 | 18 | 8 | 10 | 0 | 0 | 0 | 0 | 0 | 0 |
| 1988 | 5 | 19 | 8 | 9 | 0 | 0 | 0 | 0 | 0 | 1 |
| 1988 | 5 | 20 | 34 | 25 | 0 | 0 | 0 | 0 | 0 | 0 |
| 1988 | 5 | 22 | 87 | 42 | 0 | 0 | 0 | 0 | 0 | 0 |
| 1988 | 5 | 24 | 8 | 16 | 0 | 0 | 0 | 0 | 0 | 1 |
| 1988 | 5 | 25 | 106 | 140 | 0 | 0 | 0 | 0 | 0 | 3 |
| 1988 | 5 | 26 | 88 | 61 | 0 | 0 | 0 | 0 | 0 | 2 |
| 1988 | 5 | 27 | 45 | 45 | 0 | 0 | 0 | 0 | 0 | 2 |
| 1988 | 5 | 29 | 34 | 43 | 0 | 0 | 0 | 0 | 2 | 1 |
| 1988 | 5 | 31 | 22 | 24 | 0 | 0 | 0 | 0 | 2 | 3 |
| 1988 | 6 | 2 | 5 | 12 | 0 | 0 | 0 | 0 | 1 | 0 |
| 1988 | 6 | 3 | 7 | 0 | 0 | 0 | 0 | 0 | 0 | 0 |
| 1988 | 6 | 5 | 19 | 6 | 0 | 0 | 0 | 0 | 0 | 0 |
| 1988 | 6 | 6 | 9 | 12 | 0 | 1 | 0 | 0 | 2 | 0 |
| 1988 | 6 | 7 | 13 | 8 | 0 | 0 | 0 | 0 | 1 | 0 |
| 1988 | 6 | 8 | 7 | 5 | 0 | 0 | 0 | 0 | 3 | 1 |
| 1988 | 6 | 9 | 7 | 5 | 0 | 0 | 2 | 0 | 1 | 0 |
| 1988 | 6 | 10 | 5 | 6 | 0 | 0 | 0 | 0 | 2 | 1 |
| 1988 | 6 | 11 | 14 | 7 | 0 | 0 | 0 | 0 | 0 | 0 |
| 1988 | 6 | 12 | 3 | 5 | 0 | 0 | 0 | 0 | 0 | 0 |
| 1988 | 6 | 13 | 6 | 5 | 0 | 0 | 3 | 1 | 2 | 1 |
| 1988 | 6 | 14 | 6 | 3 | 0 | 0 | 2 | 0 | 0 | 0 |
| 1988 | 6 | 15 | 3 | 2 | 0 | 0 | 2 | 3 | 0 | 0 |
| 1988 | 6 | 16 | 1 | 0 | 0 | 0 | 0 | 0 | 1 | 0 |
| 1988 | 6 | 17 | 1 | 1 | 0 | 0 | 0 | 0 | 0 | 0 |
| 1988 | 6 | 18 | 2 | 2 | 0 | 0 | 0 | 0 | 0 | 0 |
| 1988 | 6 | 19 | 2 | 4 | 0 | 0 | 0 | 0 | 0 | 0 |
| 1988 | 6 | 21 | 2 | 1 | 0 | 0 | 3 | 2 | 0 | 0 |
| 1988 | 6 | 22 | 2 | 4 | 0 | 0 | 2 | 2 | 0 | 0 |
| 1988 | 6 | 23 | 3 | 4 | 0 | 0 | 2 | 1 | 0 | 0 |
| 1988 | 6 | 24 | 0 | 0 | 0 | 0 | 0 | 1 | 0 | 0 |
| 1988 | 6 | 25 | 2 | 3 | 0 | 0 | 2 | 0 | 0 | 0 |
| 1988 | 6 | 26 | 1 | 3 | 0 | 0 | 0 | 0 | 0 | 0 |
| 1988 | 6 | 27 | 2 | 0 | 0 | 0 | 1 | 0 | 0 | 0 |
| 1988 | 6 | 28 | 1 | 0 | 0 | 0 | 1 | 0 | 0 | 0 |
| 1988 | 6 | 30 | 2 | 0 | 0 | 0 | 0 | 0 | 0 | 0 |
| 1988 | 7 | 4 | 2 | 1 | 0 | 0 | 0 | 0 | 0 | 0 |
| 1988 | 7 | 5 | 1 | 1 | 0 | 0 | 0 | 0 | 0 | 0 |
| 1988 | 7 | 6 | 1 | 0 | 0 | 0 | 2 | 8 | 0 | 0 |
| 1988 | 7 | 7 | 3 | 1 | 0 | 0 | 0 | 8 | 0 | 0 |
| 1988 | 7 | 8 | 3 | 2 | 0 | 0 | 3 | 0 | 0 | 0 |
| 1988 | 7 | 9 | 0 | 0 | 0 | 0 | 0 | 0 | 0 | 0 |
| 1988 | 7 | 10 | 3 | 0 | 0 | 0 | 0 | 0 | 0 | 0 |
| 1988 | 7 | 12 | 1 | 1 | 0 | 0 | 0 | 0 | 0 | 0 |
| 1989 | 5 | 16 | 2 | 0 | 0 | 0 | 0 | 0 | 0 | 1 |
| 1989 | 5 | 17 | 36 | 21 | 0 | 0 | 0 | 0 | 0 | 2 |
| 1989 | 5 | 18 | 11 | 109 | 0 | 0 | 0 | 0 | 0 | 0 |
| 1989 | 5 | 19 | 39 | 28 | 0 | 0 | 0 | 0 | 1 | 13 |
| 1989 | 5 | 20 | 42 | 24 | 0 | 0 | 0 | 0 | 2 | 18 |
| 1989 | 5 | 21 | 74 | 34 | 0 | 0 | 0 | 0 | 6 | 25 |
| 1989 | 5 | 22 | 33 | 35 | 0 | 0 | 0 | 0 | 16 | 10 |
| 1989 | 5 | 23 | 13 | 71 | 0 | 0 | 0 | 0 | 5 | 9 |
| 1989 | 5 | 24 | 22 | 28 | 0 | 0 | 0 | 0 | 23 | 6 |
| 1989 | 5 | 25 | 18 | 14 | 0 | 0 | 0 | 0 | 40 | 4 |
| 1989 | 5 | 27 | 7 | 18 | 0 | 0 | 0 | 0 | 15 | 0 |
| 1989 | 5 | 29 | 12 | 16 | 0 | 0 | 0 | 0 | 9 | 1 |
| 1989 | 5 | 30 | 8 | 8 | 0 | 0 | 0 | 0 | 0 | 0 |
| 1989 | 5 | 31 | 31 | 27 | 0 | 0 | 2 | 0 | 7 | 0 |
| 1989 | 6 | 1 | 14 | 11 | 0 | 0 | 0 | 0 | 2 | 0 |
| 1989 | 6 | 2 | 15 | 14 | 0 | 0 | 1 | 1 | 1 | 0 |
| 1989 | 6 | 3 | 17 | 10 | 0 | 0 | 0 | 0 | 0 | 0 |
| 1989 | 6 | 4 | 21 | 7 | 0 | 0 | 2 | 0 | 1 | 0 |
| 1989 | 6 | 5 | 20 | 11 | 0 | 0 | 1 | 0 | 1 | 0 |
| 1989 | 6 | 6 | 15 | 10 | 0 | 0 | 0 | 0 | 0 | 0 |
| 1989 | 6 | 7 | 8 | 3 | 0 | 0 | 3 | 0 | 1 | 0 |
| 1989 | 6 | 8 | 11 | 4 | 0 | 0 | 1 | 0 | 0 | 0 |
| 1989 | 6 | 11 | 12 | 8 | 0 | 0 | 0 | 0 | 1 | 0 |
| 1989 | 6 | 12 | 10 | 7 | 0 | 0 | 0 | 0 | 0 | 0 |
| 1989 | 6 | 13 | 9 | 4 | 0 | 0 | 1 | 2 | 0 | 0 |
| 1989 | 6 | 14 | 9 | 3 | 0 | 0 | 2 | 0 | 0 | 0 |
| 1989 | 6 | 16 | 7 | 6 | 0 | 0 | 2 | 4 | 0 | 0 |
| 1989 | 6 | 17 | 3 | 0 | 0 | 0 | 2 | 1 | 0 | 0 |
| 1989 | 6 | 18 | 2 | 1 | 0 | 0 | 2 | 1 | 0 | 0 |
| 1989 | 6 | 19 | 2 | 3 | 0 | 0 | 0 | 0 | 0 | 0 |
| 1989 | 6 | 20 | 3 | 0 | 0 | 0 | 2 | 0 | 0 | 0 |
| 1989 | 6 | 21 | 1 | 0 | 0 | 0 | 0 | 0 | 0 | 0 |
| 1989 | 6 | 23 | 1 | 1 | 0 | 0 | 2 | 0 | 0 | 0 |
| 1989 | 6 | 26 | 5 | 2 | 0 | 0 | 3 | 4 | 0 | 0 |
| 1989 | 6 | 27 | 1 | 0 | 0 | 0 | 2 | 2 | 0 | 0 |
| 1989 | 6 | 29 | 7 | 3 | 0 | 0 | 2 | 1 | 0 | 0 |
| 1989 | 6 | 30 | 5 | 1 | 0 | 0 | 2 | 1 | 0 | 0 |
| 1989 | 7 | 2 | 4 | 0 | 0 | 0 | 1 | 0 | 0 | 0 |
| 1989 | 7 | 4 | 3 | 0 | 0 | 0 | 5 | 1 | 0 | 0 |
| 1989 | 7 | 7 | 2 | 1 | 0 | 0 | 2 | 0 | 0 | 0 |
| 1989 | 7 | 8 | 3 | 0 | 0 | 0 | 4 | 1 | 0 | 0 |
| 1989 | 7 | 11 | 1 | 0 | 0 | 0 | 0 | 0 | 0 | 0 |
| 1989 | 7 | 12 | 1 | 0 | 0 | 0 | 0 | 0 | 0 | 0 |
| 1989 | 7 | 15 | 1 | 0 | 0 | 0 | 0 | 0 | 0 | 0 |
| 1989 | 7 | 18 | 1 | 0 | 0 | 0 | 0 | 0 | 0 | 0 |
| 1989 | 7 | 20 | 1 | 0 | 0 | 0 | 1 | 0 | 0 | 0 |
| 1990 | 5 | 14 | 0 | 0 | 0 | 0 | 0 | 0 | 0 | 0 |
| 1990 | 5 | 16 | 1 | 2 | 0 | 0 | 0 | 0 | 1 | 1 |
| 1990 | 5 | 20 | 16 | 10 | 0 | 0 | 0 | 0 | 0 | 0 |
| 1990 | 5 | 22 | 9 | 4 | 0 | 0 | 0 | 0 | 0 | 1 |
| 1990 | 5 | 24 | 7 | 6 | 0 | 0 | 0 | 0 | 0 | 0 |
| 1990 | 5 | 26 | 1 | 3 | 0 | 0 | 0 | 0 | 0 | 1 |
| 1990 | 5 | 28 | 19 | 15 | 0 | 0 | 0 | 0 | 0 | 2 |
| 1990 | 5 | 30 | 9 | 1 | 0 | 0 | 0 | 0 | 1 | 0 |
| 1990 | 6 | 1 | 7 | 2 | 0 | 0 | 1 | 0 | 0 | 1 |
| 1990 | 6 | 3 | 25 | 11 | 0 | 0 | 1 | 0 | 0 | 0 |
| 1990 | 6 | 5 | 8 | 3 | 0 | 0 | 0 | 0 | 0 | 0 |
| 1990 | 6 | 7 | 19 | 13 | 0 | 0 | 1 | 0 | 0 | 0 |
| 1990 | 6 | 9 | 6 | 5 | 0 | 0 | 0 | 0 | 0 | 0 |
| 1990 | 6 | 11 | 11 | 18 | 0 | 0 | 0 | 0 | 0 | 0 |
| 1990 | 6 | 13 | 9 | 4 | 0 | 0 | 0 | 1 | 0 | 0 |
| 1990 | 6 | 16 | 1 | 0 | 0 | 0 | 1 | 0 | 0 | 0 |
| 1990 | 6 | 18 | 4 | 2 | 0 | 0 | 1 | 0 | 0 | 0 |
| 1990 | 6 | 21 | 0 | 1 | 0 | 0 | 2 | 1 | 0 | 0 |
| 1990 | 6 | 23 | 6 | 7 | 0 | 0 | 11 | 2 | 0 | 0 |
| 1990 | 6 | 27 | 3 | 0 | 0 | 0 | 7 | 3 | 0 | 0 |
| 1990 | 7 | 6 | 1 | 0 | 0 | 0 | 3 | 0 | 0 | 0 |
| 1990 | 7 | 9 | 2 | 0 | 0 | 0 | 6 | 0 | 0 | 0 |
| 1991 | 5 | 15 | 0 | 0 | 0 | 0 | 0 | 0 | 0 | 0 |
| 1991 | 5 | 16 | 1 | 0 | 0 | 1 | 0 | 0 | 0 | 0 |
| 1991 | 5 | 17 | 0 | 0 | 0 | 0 | 0 | 0 | 0 | 0 |
| 1991 | 5 | 18 | 0 | 0 | 1 | 0 | 0 | 0 | 0 | 0 |
| 1991 | 5 | 19 | 1 | 0 | 0 | 0 | 0 | 0 | 0 | 0 |
| 1991 | 5 | 20 | 3 | 1 | 0 | 1 | 1 | 0 | 0 | 0 |
| 1991 | 5 | 21 | 9 | 1 | 0 | 0 | 0 | 0 | 1 | 0 |
| 1991 | 5 | 22 | 10 | 5 | 0 | 0 | 0 | 0 | 1 | 0 |
| 1991 | 5 | 23 | 13 | 3 | 0 | 0 | 0 | 0 | 0 | 1 |
| 1991 | 5 | 24 | 14 | 8 | 0 | 0 | 0 | 0 | 1 | 1 |
| 1991 | 5 | 28 | 35 | 45 | 0 | 0 | 0 | 0 | 2 | 7 |
| 1991 | 5 | 29 | 20 | 47 | 0 | 0 | 3 | 0 | 5 | 3 |
| 1991 | 5 | 30 | 9 | 11 | 0 | 0 | 0 | 0 | 5 | 3 |
| 1991 | 5 | 31 | 4 | 7 | 0 | 0 | 5 | 0 | 3 | 0 |
| 1991 | 6 | 1 | 11 | 11 | 0 | 0 | 7 | 8 | 2 | 1 |
| 1991 | 6 | 3 | 1 | 5 | 0 | 0 | 5 | 2 | 0 | 2 |
| 1991 | 6 | 4 | 2 | 3 | 0 | 0 | 3 | 0 | 2 | 1 |
| 1991 | 6 | 5 | 28 | 29 | 0 | 0 | 25 | 4 | 2 | 0 |
| 1991 | 6 | 6 | 23 | 13 | 0 | 0 | 1 | 0 | 1 | 0 |
| 1991 | 6 | 7 | 6 | 13 | 0 | 0 | 0 | 0 | 0 | 0 |
| 1991 | 6 | 8 | 8 | 9 | 0 | 0 | 2 | 0 | 0 | 0 |
| 1991 | 6 | 10 | 8 | 18 | 0 | 0 | 0 | 0 | 0 | 0 |
| 1991 | 6 | 11 | 15 | 12 | 0 | 0 | 1 | 0 | 0 | 0 |
| 1991 | 6 | 12 | 12 | 10 | 0 | 0 | 0 | 0 | 0 | 0 |
| 1991 | 6 | 13 | 10 | 9 | 0 | 0 | 1 | 0 | 0 | 0 |
| 1991 | 6 | 14 | 8 | 5 | 0 | 0 | 1 | 2 | 0 | 0 |
| 1991 | 6 | 15 | 1 | 10 | 0 | 0 | 3 | 5 | 0 | 0 |
| 1991 | 6 | 16 | 1 | 0 | 0 | 0 | 0 | 1 | 0 | 0 |
| 1991 | 6 | 17 | 8 | 1 | 0 | 0 | 0 | 0 | 0 | 0 |
| 1991 | 6 | 18 | 3 | 5 | 0 | 0 | 2 | 0 | 0 | 0 |
| 1991 | 6 | 19 | 4 | 2 | 0 | 0 | 3 | 4 | 0 | 0 |
| 1991 | 6 | 20 | 3 | 1 | 0 | 0 | 4 | 0 | 0 | 0 |
| 1991 | 6 | 21 | 4 | 5 | 0 | 0 | 1 | 2 | 0 | 0 |
| 1991 | 6 | 22 | 0 | 2 | 0 | 0 | 3 | 5 | 0 | 0 |
| 1991 | 6 | 24 | 12 | 3 | 0 | 0 | 7 | 5 | 0 | 0 |
| 1991 | 6 | 25 | 3 | 1 | 0 | 0 | 3 | 2 | 0 | 0 |
| 1991 | 6 | 26 | 3 | 2 | 0 | 0 | 0 | 2 | 0 | 0 |
| 1991 | 6 | 27 | 2 | 0 | 0 | 0 | 5 | 8 | 0 | 0 |
| 1991 | 6 | 28 | 2 | 3 | 0 | 0 | 0 | 0 | 0 | 0 |
| 1991 | 6 | 29 | 2 | 1 | 0 | 0 | 3 | 4 | 0 | 0 |
| 1991 | 7 | 1 | 1 | 0 | 0 | 0 | 1 | 2 | 0 | 0 |
| 1991 | 7 | 3 | 0 | 3 | 0 | 0 | 0 | 0 | 0 | 0 |
| 1991 | 7 | 5 | 0 | 2 | 0 | 0 | 7 | 7 | 0 | 0 |
| 1991 | 7 | 7 | 0 | 1 | 0 | 0 | 0 | 0 | 0 | 0 |
| 1991 | 7 | 9 | 1 | 0 | 0 | 0 | 0 | 0 | 0 | 0 |
| 1991 | 7 | 11 | 1 | 0 | 0 | 0 | 0 | 0 | 0 | 0 |
| 1992 | 5 | 13 | 0 | 0 | 1 | 1 | 0 | 0 | 0 | 0 |
| 1992 | 5 | 19 | 1 | 0 | 0 | 0 | 0 | 0 | 0 | 0 |
| 1992 | 5 | 20 | 2 | 0 | 1 | 0 | 0 | 0 | 0 | 1 |
| 1992 | 5 | 21 | 2 | 2 | 0 | 0 | 0 | 0 | 1 | 3 |
| 1992 | 5 | 22 | 5 | 5 | 0 | 0 | 0 | 0 | 0 | 4 |
| 1992 | 5 | 23 | 9 | 2 | 0 | 0 | 0 | 0 | 6 | 7 |
| 1992 | 5 | 24 | 3 | 9 | 0 | 0 | 0 | 0 | 1 | 2 |
| 1992 | 5 | 25 | 2 | 2 | 0 | 0 | 0 | 0 | 1 | 0 |
| 1992 | 5 | 26 | 1 | 1 | 0 | 0 | 0 | 0 | 1 | 1 |
| 1992 | 5 | 27 | 0 | 4 | 0 | 0 | 0 | 0 | 1 | 0 |
| 1992 | 5 | 28 | 3 | 3 | 0 | 0 | 0 | 0 | 1 | 2 |
| 1992 | 5 | 29 | 32 | 14 | 0 | 0 | 0 | 0 | 15 | 11 |
| 1992 | 5 | 30 | 2 | 8 | 0 | 0 | 0 | 0 | 11 | 5 |
| 1992 | 5 | 31 | 17 | 39 | 0 | 0 | 0 | 0 | 6 | 1 |
| 1992 | 6 | 1 | 28 | 15 | 0 | 0 | 0 | 0 | 11 | 3 |
| 1992 | 6 | 2 | 18 | 23 | 0 | 0 | 0 | 0 | 7 | 0 |
| 1992 | 6 | 3 | 9 | 8 | 0 | 0 | 0 | 0 | 7 | 1 |
| 1992 | 6 | 4 | 23 | 30 | 0 | 0 | 0 | 0 | 1 | 0 |
| 1992 | 6 | 5 | 5 | 19 | 0 | 0 | 0 | 0 | 1 | 0 |
| 1992 | 6 | 6 | 42 | 22 | 0 | 0 | 0 | 0 | 0 | 0 |
| 1992 | 6 | 7 | 24 | 21 | 0 | 0 | 0 | 0 | 0 | 0 |
| 1992 | 6 | 8 | 25 | 30 | 0 | 0 | 0 | 0 | 3 | 0 |
| 1992 | 6 | 9 | 12 | 11 | 0 | 0 | 0 | 0 | 0 | 0 |
| 1992 | 6 | 10 | 18 | 17 | 0 | 0 | 0 | 0 | 0 | 0 |
| 1992 | 6 | 11 | 20 | 36 | 0 | 0 | 0 | 0 | 0 | 0 |
| 1992 | 6 | 12 | 7 | 15 | 0 | 0 | 0 | 0 | 0 | 0 |
| 1992 | 6 | 13 | 18 | 9 | 0 | 0 | 0 | 0 | 0 | 0 |
| 1992 | 6 | 14 | 6 | 7 | 0 | 0 | 1 | 1 | 0 | 0 |
| 1992 | 6 | 15 | 11 | 39 | 0 | 0 | 0 | 0 | 0 | 0 |
| 1992 | 6 | 16 | 7 | 5 | 0 | 0 | 0 | 1 | 0 | 0 |
| 1992 | 6 | 17 | 10 | 5 | 0 | 0 | 0 | 0 | 0 | 0 |
| 1992 | 6 | 18 | 2 | 3 | 0 | 0 | 1 | 1 | 0 | 0 |
| 1992 | 6 | 19 | 7 | 7 | 0 | 0 | 0 | 1 | 0 | 0 |
| 1992 | 6 | 20 | 2 | 2 | 0 | 0 | 3 | 0 | 0 | 0 |
| 1992 | 6 | 21 | 2 | 0 | 0 | 0 | 1 | 2 | 0 | 0 |
| 1992 | 6 | 22 | 2 | 4 | 0 | 0 | 1 | 1 | 0 | 0 |
| 1992 | 6 | 23 | 5 | 4 | 0 | 0 | 0 | 0 | 0 | 0 |
| 1992 | 6 | 25 | 3 | 14 | 0 | 0 | 0 | 0 | 0 | 0 |
| 1992 | 6 | 26 | 3 | 5 | 0 | 0 | 0 | 0 | 0 | 0 |
| 1992 | 6 | 27 | 3 | 0 | 0 | 0 | 0 | 0 | 0 | 0 |
| 1992 | 6 | 29 | 4 | 2 | 0 | 0 | 0 | 0 | 0 | 0 |
| 1992 | 6 | 30 | 2 | 3 | 0 | 0 | 6 | 4 | 0 | 0 |
| 1992 | 7 | 3 | 0 | 1 | 0 | 0 | 14 | 8 | 0 | 0 |
| 1992 | 7 | 5 | 6 | 0 | 0 | 0 | 1 | 3 | 0 | 0 |
| 1992 | 7 | 7 | 1 | 1 | 0 | 0 | 3 | 2 | 0 | 0 |
| 1992 | 7 | 9 | 7 | 2 | 0 | 0 | 7 | 8 | 0 | 0 |
| 1992 | 7 | 11 | 2 | 4 | 0 | 0 | 3 | 2 | 0 | 0 |
| 1992 | 7 | 13 | 2 | 8 | 0 | 0 | 1 | 2 | 0 | 0 |
| 1992 | 7 | 15 | 3 | 7 | 0 | 0 | 1 | 1 | 0 | 0 |
| 1993 | 5 | 12 | 1 | 0 | 0 | 0 | 0 | 0 | 0 | 0 |
| 1993 | 5 | 13 | 0 | 0 | 0 | 0 | 0 | 0 | 0 | 0 |
| 1993 | 5 | 14 | 2 | 0 | 0 | 0 | 0 | 0 | 0 | 0 |
| 1993 | 5 | 15 | 0 | 0 | 0 | 0 | 0 | 0 | 0 | 0 |
| 1993 | 5 | 16 | 0 | 0 | 0 | 0 | 0 | 0 | 0 | 0 |
| 1993 | 5 | 17 | 1 | 0 | 0 | 0 | 0 | 0 | 0 | 0 |
| 1993 | 5 | 18 | 1 | 0 | 0 | 0 | 0 | 0 | 0 | 0 |
| 1993 | 5 | 19 | 0 | 1 | 0 | 0 | 0 | 0 | 0 | 0 |
| 1993 | 5 | 20 | 1 | 0 | 0 | 1 | 0 | 0 | 0 | 0 |
| 1993 | 5 | 21 | 0 | 0 | 0 | 1 | 0 | 0 | 0 | 0 |
| 1993 | 5 | 22 | 1 | 0 | 0 | 0 | 0 | 0 | 0 | 0 |
| 1993 | 5 | 23 | 0 | 0 | 0 | 0 | 0 | 0 | 0 | 0 |
| 1993 | 5 | 24 | 18 | 8 | 0 | 0 | 0 | 0 | 1 | 5 |
| 1993 | 5 | 25 | 27 | 13 | 0 | 0 | 0 | 0 | 0 | 9 |
| 1993 | 5 | 26 | 27 | 13 | 0 | 0 | 0 | 0 | 1 | 17 |
| 1993 | 5 | 27 | 28 | 25 | 0 | 0 | 0 | 0 | 3 | 31 |
| 1993 | 5 | 28 | 29 | 14 | 0 | 0 | 0 | 0 | 5 | 23 |
| 1993 | 5 | 29 | 23 | 23 | 0 | 0 | 0 | 0 | 13 | 23 |
| 1993 | 5 | 30 | 4 | 2 | 0 | 0 | 0 | 0 | 5 | 5 |
| 1993 | 5 | 31 | 42 | 20 | 0 | 0 | 0 | 0 | 18 | 20 |
| 1993 | 6 | 1 | 38 | 18 | 0 | 0 | 0 | 0 | 46 | 19 |
| 1993 | 6 | 2 | 47 | 36 | 0 | 0 | 0 | 0 | 17 | 7 |
| 1993 | 6 | 3 | 5 | 6 | 0 | 0 | 0 | 0 | 7 | 6 |
| 1993 | 6 | 4 | 1 | 0 | 0 | 0 | 0 | 0 | 6 | 1 |
| 1993 | 6 | 5 | 69 | 37 | 0 | 0 | 0 | 0 | 9 | 4 |
| 1993 | 6 | 6 | 32 | 19 | 0 | 0 | 0 | 0 | 12 | 4 |
| 1993 | 6 | 7 | 34 | 21 | 0 | 0 | 0 | 0 | 6 | 1 |
| 1993 | 6 | 8 | 22 | 22 | 0 | 0 | 0 | 0 | 4 | 0 |
| 1993 | 6 | 9 | 9 | 10 | 0 | 0 | 0 | 0 | 3 | 1 |
| 1993 | 6 | 10 | 40 | 16 | 0 | 0 | 0 | 0 | 1 | 0 |
| 1993 | 6 | 11 | 15 | 16 | 0 | 0 | 0 | 0 | 5 | 0 |
| 1993 | 6 | 12 | 2 | 4 | 0 | 0 | 0 | 0 | 6 | 0 |
| 1993 | 6 | 13 | 3 | 6 | 0 | 0 | 0 | 0 | 2 | 0 |
| 1993 | 6 | 14 | 11 | 15 | 0 | 0 | 0 | 0 | 3 | 0 |
| 1993 | 6 | 15 | 8 | 5 | 0 | 0 | 0 | 0 | 2 | 0 |
| 1993 | 6 | 16 | 7 | 4 | 0 | 0 | 1 | 0 | 3 | 1 |
| 1993 | 6 | 17 | 4 | 3 | 0 | 0 | 3 | 3 | 0 | 0 |
| 1993 | 6 | 18 | 7 | 4 | 0 | 0 | 3 | 0 | 0 | 0 |
| 1993 | 6 | 19 | 6 | 5 | 0 | 0 | 2 | 0 | 1 | 0 |
| 1993 | 6 | 20 | 0 | 3 | 0 | 0 | 1 | 0 | 0 | 0 |
| 1993 | 6 | 21 | 3 | 1 | 0 | 0 | 1 | 0 | 0 | 0 |
| 1993 | 6 | 22 | 1 | 3 | 0 | 0 | 0 | 1 | 0 | 0 |
| 1993 | 6 | 23 | 1 | 0 | 0 | 0 | 1 | 0 | 0 | 0 |
| 1993 | 6 | 24 | 3 | 0 | 0 | 0 | 2 | 2 | 0 | 0 |
| 1993 | 6 | 26 | 3 | 0 | 0 | 0 | 11 | 3 | 0 | 0 |
| 1993 | 6 | 28 | 5 | 1 | 0 | 0 | 4 | 1 | 0 | 0 |
| 1993 | 7 | 2 | 3 | 2 | 0 | 0 | 6 | 6 | 0 | 0 |
| 1993 | 7 | 3 | 2 | 1 | 0 | 0 | 0 | 3 | 0 | 0 |
| 1993 | 7 | 5 | 3 | 0 | 0 | 0 | 6 | 1 | 0 | 0 |
| 1993 | 7 | 6 | 1 | 0 | 0 | 0 | 1 | 1 | 0 | 0 |
| 1993 | 7 | 7 | 1 | 0 | 0 | 0 | 1 | 2 | 0 | 0 |
| 1993 | 7 | 8 | 0 | 0 | 0 | 0 | 1 | 1 | 0 | 0 |
| 1993 | 7 | 9 | 0 | 0 | 0 | 0 | 0 | 0 | 0 | 0 |
| 1993 | 7 | 10 | 0 | 0 | 0 | 0 | 1 | 0 | 0 | 0 |
| 1993 | 7 | 15 | 1 | 0 | 0 | 0 | 1 | 0 | 0 | 0 |
| 1993 | 7 | 16 | 0 | 0 | 0 | 0 | 1 | 0 | 0 | 0 |
| 1993 | 7 | 17 | 0 | 0 | 0 | 0 | 0 | 0 | 0 | 0 |
| 1993 | 7 | 19 | 2 | 0 | 0 | 0 | 0 | 0 | 0 | 0 |
| 1993 | 7 | 20 | 0 | 0 | 0 | 0 | 0 | 0 | 0 | 0 |
| 1993 | 7 | 21 | 0 | 0 | 0 | 0 | 0 | 0 | 0 | 0 |
| 1993 | 7 | 22 | 0 | 0 | 0 | 0 | 0 | 0 | 0 | 0 |
| 1993 | 7 | 23 | 0 | 0 | 0 | 0 | 0 | 0 | 0 | 0 |
| 1994 | 5 | 9 | 0 | 0 | 0 | 0 | 0 | 0 | 0 | 0 |
| 1994 | 5 | 10 | 1 | 0 | 0 | 0 | 0 | 0 | 0 | 0 |
| 1994 | 5 | 12 | 4 | 0 | 0 | 0 | 0 | 0 | 0 | 0 |
| 1994 | 5 | 13 | 4 | 0 | 0 | 0 | 0 | 0 | 0 | 0 |
| 1994 | 5 | 17 | 1 | 0 | 0 | 0 | 0 | 0 | 0 | 0 |
| 1994 | 5 | 18 | 0 | 0 | 0 | 0 | 0 | 0 | 0 | 3 |
| 1994 | 5 | 19 | 0 | 0 | 0 | 0 | 0 | 0 | 0 | 0 |
| 1994 | 5 | 20 | 0 | 0 | 0 | 0 | 0 | 0 | 0 | 0 |
| 1994 | 5 | 21 | 0 | 0 | 0 | 0 | 0 | 0 | 0 | 0 |
| 1994 | 5 | 22 | 0 | 0 | 0 | 0 | 0 | 0 | 0 | 0 |
| 1994 | 5 | 23 | 2 | 3 | 0 | 0 | 0 | 0 | 0 | 2 |
| 1994 | 5 | 24 | 11 | 7 | 0 | 0 | 0 | 0 | 5 | 8 |
| 1994 | 5 | 26 | 0 | 0 | 0 | 0 | 0 | 0 | 0 | 0 |
| 1994 | 5 | 27 | 13 | 14 | 0 | 0 | 0 | 0 | 1 | 0 |
| 1994 | 5 | 28 | 11 | 12 | 0 | 0 | 1 | 1 | 3 | 0 |
| 1994 | 5 | 29 | 21 | 32 | 0 | 0 | 0 | 0 | 5 | 3 |
| 1994 | 5 | 30 | 36 | 65 | 0 | 0 | 0 | 0 | 16 | 10 |
| 1994 | 5 | 31 | 15 | 16 | 0 | 0 | 0 | 0 | 5 | 0 |
| 1994 | 6 | 1 | 18 | 19 | 0 | 0 | 0 | 0 | 17 | 6 |
| 1994 | 6 | 2 | 11 | 17 | 0 | 0 | 0 | 0 | 0 | 0 |
| 1994 | 6 | 3 | 6 | 11 | 0 | 0 | 0 | 0 | 0 | 0 |
| 1994 | 6 | 4 | 7 | 5 | 0 | 0 | 0 | 0 | 3 | 3 |
| 1994 | 6 | 5 | 7 | 8 | 0 | 0 | 0 | 0 | 0 | 0 |
| 1994 | 6 | 6 | 11 | 15 | 0 | 0 | 0 | 0 | 0 | 0 |
| 1994 | 6 | 7 | 6 | 16 | 0 | 0 | 0 | 0 | 1 | 0 |
| 1994 | 6 | 8 | 27 | 57 | 0 | 0 | 0 | 0 | 2 | 0 |
| 1994 | 6 | 9 | 7 | 12 | 0 | 0 | 0 | 0 | 0 | 0 |
| 1994 | 6 | 10 | 10 | 8 | 0 | 0 | 0 | 0 | 1 | 0 |
| 1994 | 6 | 11 | 9 | 11 | 0 | 0 | 0 | 1 | 0 | 0 |
| 1994 | 6 | 13 | 18 | 9 | 0 | 0 | 1 | 0 | 0 | 0 |
| 1994 | 6 | 14 | 9 | 12 | 0 | 0 | 0 | 1 | 0 | 0 |
| 1994 | 6 | 15 | 15 | 22 | 0 | 0 | 0 | 1 | 0 | 0 |
| 1994 | 6 | 16 | 10 | 5 | 0 | 0 | 2 | 4 | 0 | 0 |
| 1994 | 6 | 17 | 6 | 6 | 0 | 0 | 1 | 1 | 0 | 0 |
| 1994 | 6 | 20 | 0 | 0 | 0 | 0 | 0 | 0 | 0 | 0 |
| 1994 | 6 | 21 | 15 | 9 | 0 | 0 | 1 | 2 | 0 | 0 |
| 1994 | 6 | 22 | 9 | 2 | 0 | 0 | 0 | 0 | 0 | 0 |
| 1994 | 6 | 25 | 1 | 2 | 0 | 0 | 2 | 3 | 0 | 0 |
| 1994 | 6 | 28 | 12 | 13 | 0 | 0 | 20 | 4 | 0 | 0 |
| 1994 | 7 | 1 | 7 | 3 | 0 | 0 | 6 | 6 | 0 | 0 |
| 1994 | 7 | 4 | 4 | 9 | 0 | 0 | 11 | 5 | 0 | 0 |
| 1994 | 7 | 5 | 4 | 7 | 0 | 0 | 12 | 8 | 0 | 0 |
| 1994 | 7 | 6 | 2 | 7 | 0 | 0 | 8 | 2 | 0 | 0 |
| 1994 | 7 | 7 | 7 | 20 | 0 | 0 | 1 | 0 | 1 | 0 |
| 1994 | 7 | 8 | 5 | 2 | 0 | 0 | 0 | 1 | 0 | 0 |
| 1994 | 7 | 9 | 2 | 0 | 0 | 0 | 0 | 0 | 0 | 0 |
| 1994 | 7 | 10 | 3 | 3 | 0 | 0 | 0 | 1 | 0 | 0 |
| 1994 | 7 | 11 | 2 | 0 | 0 | 0 | 0 | 0 | 0 | 0 |
| 1994 | 7 | 12 | 4 | 4 | 0 | 0 | 1 | 1 | 0 | 0 |
| 1994 | 7 | 13 | 0 | 0 | 0 | 0 | 0 | 0 | 0 | 0 |
| 1995 | 5 | 16 | 0 | 0 | 0 | 0 | 0 | 0 | 0 | 0 |
| 1995 | 5 | 17 | 3 | 0 | 0 | 3 | 0 | 0 | 0 | 0 |
| 1995 | 5 | 18 | 8 | 3 | 0 | 3 | 0 | 0 | 0 | 0 |
| 1995 | 5 | 19 | 2 | 0 | 0 | 2 | 0 | 0 | 0 | 0 |
| 1995 | 5 | 22 | 0 | 0 | 0 | 1 | 0 | 0 | 0 | 0 |
| 1995 | 5 | 23 | 1 | 1 | 0 | 0 | 0 | 0 | 0 | 0 |
| 1995 | 5 | 26 | 4 | 0 | 0 | 0 | 0 | 0 | 0 | 2 |
| 1995 | 5 | 29 | 12 | 4 | 0 | 0 | 0 | 0 | 1 | 13 |
| 1995 | 5 | 30 | 12 | 13 | 0 | 0 | 0 | 0 | 7 | 23 |
| 1995 | 5 | 31 | 17 | 5 | 0 | 0 | 0 | 0 | 23 | 50 |
| 1995 | 6 | 1 | 7 | 4 | 0 | 0 | 1 | 0 | 22 | 31 |
| 1995 | 6 | 2 | 7 | 11 | 0 | 0 | 0 | 0 | 23 | 18 |
| 1995 | 6 | 5 | 8 | 3 | 0 | 0 | 0 | 0 | 19 | 19 |
| 1995 | 6 | 7 | 15 | 15 | 0 | 0 | 1 | 0 | 45 | 24 |
| 1995 | 6 | 8 | 10 | 6 | 0 | 0 | 1 | 0 | 10 | 6 |
| 1995 | 6 | 9 | 7 | 5 | 0 | 0 | 0 | 0 | 2 | 1 |
| 1995 | 6 | 12 | 16 | 22 | 0 | 0 | 1 | 0 | 10 | 1 |
| 1995 | 6 | 13 | 2 | 5 | 0 | 0 | 0 | 0 | 0 | 0 |
| 1995 | 6 | 14 | 12 | 5 | 0 | 0 | 1 | 1 | 1 | 0 |
| 1995 | 6 | 16 | 22 | 6 | 0 | 0 | 1 | 1 | 0 | 0 |
| 1995 | 6 | 19 | 12 | 4 | 0 | 0 | 0 | 0 | 1 | 0 |
| 1995 | 6 | 21 | 12 | 10 | 0 | 0 | 1 | 0 | 1 | 0 |
| 1995 | 6 | 28 | 12 | 3 | 0 | 0 | 3 | 0 | 1 | 1 |
| 1995 | 6 | 29 | 4 | 1 | 0 | 0 | 1 | 1 | 0 | 0 |
| 1995 | 6 | 30 | 2 | 2 | 0 | 0 | 2 | 2 | 0 | 0 |
| 1995 | 7 | 4 | 5 | 2 | 0 | 0 | 7 | 5 | 0 | 0 |
| 1995 | 7 | 6 | 3 | 0 | 0 | 0 | 3 | 1 | 0 | 0 |
| 1995 | 7 | 10 | 3 | 1 | 0 | 0 | 5 | 4 | 0 | 0 |
| 1995 | 7 | 12 | 3 | 4 | 0 | 0 | 5 | 0 | 0 | 0 |
| 1995 | 7 | 14 | 2 | 2 | 0 | 0 | 3 | 2 | 0 | 0 |
| 1995 | 7 | 18 | 0 | 1 | 0 | 0 | 0 | 1 | 0 | 0 |
| 1995 | 7 | 20 | 0 | 1 | 0 | 0 | 1 | 0 | 0 | 0 |
| 1995 | 7 | 27 | 0 | 0 | 0 | 0 | 0 | 0 | 0 | 0 |
| 1996 | 5 | 12 | 0 | 0 | 0 | 0 | 0 | 0 | 0 | 0 |
| 1996 | 5 | 16 | 92 | 68 | 0 | 0 | 0 | 0 | 5 | 56 |
| 1996 | 5 | 20 | 122 | 65 | 1 | 0 | 0 | 0 | 164 | 167 |
| 1996 | 5 | 24 | 40 | 18 | 1 | 3 | 0 | 0 | 57 | 23 |
| 1996 | 5 | 28 | 101 | 69 | 4 | 1 | 0 | 0 | 42 | 15 |
| 1996 | 6 | 1 | 96 | 87 | 0 | 0 | 0 | 0 | 15 | 3 |
| 1996 | 6 | 5 | 14 | 17 | 0 | 0 | 1 | 0 | 1 | 0 |
| 1996 | 6 | 9 | 111 | 77 | 0 | 0 | 2 | 2 | 2 | 1 |
| 1996 | 6 | 13 | 87 | 62 | 0 | 0 | 4 | 9 | 0 | 0 |
| 1996 | 6 | 17 | 77 | 56 | 0 | 0 | 17 | 12 | 0 | 0 |
| 1996 | 6 | 21 | 28 | 29 | 0 | 0 | 10 | 10 | 0 | 0 |
| 1996 | 6 | 25 | 57 | 47 | 0 | 0 | 45 | 34 | 0 | 0 |
| 1996 | 6 | 29 | 15 | 12 | 0 | 0 | 36 | 27 | 0 | 0 |
| 1996 | 7 | 3 | 28 | 21 | 0 | 0 | 106 | 61 | 0 | 0 |
| 1996 | 7 | 7 | 19 | 7 | 0 | 0 | 40 | 26 | 0 | 0 |
| 1996 | 7 | 11 | 5 | 3 | 0 | 0 | 11 | 8 | 0 | 0 |
| 1996 | 7 | 15 | 11 | 5 | 0 | 0 | 3 | 4 | 0 | 0 |
| 1996 | 7 | 19 | 2 | 3 | 0 | 0 | 0 | 0 | 0 | 0 |
| 1996 | 7 | 23 | 0 | 0 | 0 | 0 | 0 | 0 | 0 | 0 |
| 1996 | 7 | 27 | 2 | 1 | 0 | 0 | 1 | 0 | 0 | 0 |
| 1996 | 7 | 31 | 0 | 0 | 0 | 0 | 0 | 1 | 0 | 0 |
| 1997 | 5 | 12 | 0 | 0 | 0 | 0 | 0 | 0 | 0 | 0 |
| 1997 | 5 | 16 | 0 | 0 | 0 | 0 | 0 | 0 | 0 | 0 |
| 1997 | 5 | 20 | 25 | 17 | 1 | 1 | 0 | 0 | 1 | 3 |
| 1997 | 5 | 24 | 12 | 14 | 1 | 1 | 0 | 0 | 1 | 2 |
| 1997 | 5 | 28 | 124 | 74 | 1 | 3 | 0 | 0 | 50 | 77 |
| 1997 | 6 | 1 | 0 | 0 | 0 | 0 | 0 | 0 | 0 | 0 |
| 1997 | 6 | 5 | 139 | 79 | 4 | 5 | 0 | 0 | 91 | 25 |
| 1997 | 6 | 9 | 247 | 170 | 2 | 0 | 7 | 2 | 3 | 2 |
| 1997 | 6 | 13 | 170 | 152 | 0 | 0 | 3 | 3 | 0 | 0 |
| 1997 | 6 | 17 | 111 | 67 | 0 | 0 | 17 | 16 | 0 | 0 |
| 1997 | 6 | 21 | 46 | 40 | 0 | 0 | 34 | 40 | 0 | 0 |
| 1997 | 6 | 25 | 30 | 21 | 0 | 0 | 25 | 33 | 0 | 0 |
| 1997 | 6 | 29 | 27 | 17 | 0 | 0 | 45 | 45 | 0 | 0 |
| 1997 | 7 | 3 | 0 | 0 | 0 | 0 | 0 | 0 | 0 | 0 |
| 1997 | 7 | 7 | 20 | 10 | 0 | 0 | 103 | 63 | 0 | 0 |
| 1997 | 7 | 11 | 0 | 0 | 0 | 0 | 0 | 0 | 0 | 0 |
| 1997 | 7 | 15 | 10 | 3 | 0 | 0 | 49 | 24 | 0 | 0 |
| 1997 | 7 | 19 | 13 | 7 | 0 | 0 | 7 | 5 | 0 | 0 |
| 1997 | 7 | 23 | 8 | 3 | 0 | 0 | 2 | 1 | 0 | 0 |
| 1997 | 7 | 27 | 2 | 2 | 0 | 0 | 0 | 0 | 0 | 0 |
| 1997 | 7 | 31 | 0 | 0 | 0 | 0 | 0 | 0 | 0 | 0 |
| 1998 | 5 | 12 | 0 | 0 | 0 | 0 | 0 | 0 | 0 | 0 |
| 1998 | 5 | 16 | 0 | 0 | 0 | 0 | 0 | 0 | 0 | 0 |
| 1998 | 5 | 20 | 0 | 0 | 0 | 0 | 0 | 0 | 0 | 0 |
| 1998 | 5 | 24 | 3 | 0 | 0 | 1 | 0 | 0 | 2 | 5 |
| 1998 | 5 | 28 | 4 | 4 | 0 | 0 | 0 | 0 | 6 | 13 |
| 1998 | 6 | 1 | 141 | 98 | 3 | 14 | 0 | 0 | 131 | 158 |
| 1998 | 6 | 5 | 119 | 77 | 11 | 16 | 0 | 0 | 131 | 29 |
| 1998 | 6 | 9 | 66 | 34 | 8 | 14 | 0 | 0 | 9 | 0 |
| 1998 | 6 | 13 | 63 | 62 | 2 | 1 | 2 | 3 | 0 | 0 |
| 1998 | 6 | 17 | 67 | 54 | 1 | 0 | 6 | 4 | 4 | 2 |
| 1998 | 6 | 21 | 136 | 102 | 0 | 0 | 25 | 18 | 0 | 0 |
| 1998 | 6 | 25 | 0 | 0 | 0 | 0 | 0 | 0 | 0 | 0 |
| 1998 | 6 | 29 | 63 | 36 | 0 | 0 | 55 | 47 | 1 | 0 |
| 1998 | 7 | 3 | 0 | 0 | 0 | 0 | 0 | 0 | 0 | 0 |
| 1998 | 7 | 7 | 27 | 9 | 0 | 0 | 63 | 39 | 0 | 0 |
| 1998 | 7 | 11 | 12 | 9 | 0 | 0 | 15 | 9 | 0 | 0 |
| 1998 | 7 | 15 | 7 | 3 | 0 | 0 | 2 | 4 | 0 | 0 |
| 1998 | 7 | 19 | 6 | 5 | 0 | 0 | 1 | 0 | 0 | 0 |
| 1998 | 7 | 23 | 0 | 0 | 0 | 0 | 0 | 0 | 0 | 0 |
| 1998 | 7 | 27 | 0 | 0 | 0 | 0 | 0 | 0 | 0 | 0 |
| 1998 | 7 | 31 | 0 | 0 | 0 | 0 | 0 | 0 | 0 | 0 |
| 1999 | 5 | 12 | 0 | 0 | 0 | 0 | 0 | 0 | 0 | 0 |
| 1999 | 5 | 16 | 0 | 1 | 0 | 0 | 0 | 0 | 0 | 1 |
| 1999 | 5 | 20 | 0 | 1 | 0 | 0 | 0 | 0 | 0 | 1 |
| 1999 | 5 | 24 | 6 | 3 | 0 | 0 | 0 | 0 | 2 | 10 |
| 1999 | 5 | 28 | 23 | 18 | 1 | 2 | 0 | 0 | 57 | 107 |
| 1999 | 6 | 1 | 16 | 16 | 1 | 4 | 0 | 0 | 18 | 13 |
| 1999 | 6 | 5 | 20 | 11 | 1 | 4 | 0 | 0 | 9 | 1 |
| 1999 | 6 | 9 | 32 | 17 | 0 | 0 | 1 | 0 | 1 | 0 |
| 1999 | 6 | 13 | 28 | 27 | 0 | 0 | 2 | 3 | 1 | 1 |
| 1999 | 6 | 17 | 0 | 0 | 0 | 0 | 0 | 0 | 0 | 0 |
| 1999 | 6 | 21 | 5 | 4 | 0 | 0 | 5 | 5 | 0 | 0 |
| 1999 | 6 | 25 | 12 | 12 | 0 | 0 | 10 | 2 | 0 | 0 |
| 1999 | 6 | 29 | 10 | 14 | 0 | 0 | 34 | 28 | 0 | 0 |
| 1999 | 7 | 3 | 3 | 2 | 0 | 0 | 36 | 17 | 0 | 0 |
| 1999 | 7 | 7 | 0 | 0 | 0 | 0 | 0 | 0 | 0 | 0 |
| 1999 | 7 | 11 | 13 | 4 | 0 | 0 | 10 | 7 | 0 | 0 |
| 1999 | 7 | 15 | 0 | 0 | 0 | 0 | 0 | 0 | 0 | 0 |
| 1999 | 7 | 19 | 2 | 0 | 0 | 0 | 4 | 1 | 0 | 0 |
| 1999 | 7 | 23 | 2 | 0 | 0 | 0 | 1 | 0 | 0 | 0 |
| 1999 | 7 | 27 | 0 | 0 | 0 | 0 | 0 | 0 | 0 | 0 |
| 1999 | 7 | 31 | 0 | 0 | 0 | 0 | 0 | 0 | 0 | 0 |
| 2000 | 5 | 12 | 0 | 0 | 0 | 0 | 0 | 0 | 0 | 0 |
| 2000 | 5 | 16 | 0 | 1 | 0 | 0 | 0 | 0 | 0 | 0 |
| 2000 | 5 | 20 | 4 | 3 | 0 | 0 | 0 | 0 | 1 | 4 |
| 2000 | 5 | 24 | 10 | 6 | 0 | 0 | 0 | 0 | 12 | 9 |
| 2000 | 5 | 28 | 0 | 0 | 0 | 0 | 0 | 0 | 0 | 0 |
| 2000 | 6 | 1 | 13 | 9 | 0 | 0 | 0 | 0 | 6 | 5 |
| 2000 | 6 | 5 | 9 | 12 | 0 | 0 | 0 | 0 | 4 | 2 |
| 2000 | 6 | 9 | 0 | 0 | 0 | 0 | 0 | 0 | 0 | 0 |
| 2000 | 6 | 13 | 0 | 3 | 0 | 0 | 0 | 0 | 0 | 1 |
| 2000 | 6 | 17 | 5 | 4 | 0 | 0 | 1 | 2 | 2 | 1 |
| 2000 | 6 | 21 | 3 | 5 | 0 | 0 | 11 | 4 | 0 | 0 |
| 2000 | 6 | 25 | 0 | 0 | 0 | 0 | 0 | 0 | 0 | 0 |
| 2000 | 6 | 29 | 4 | 3 | 0 | 0 | 8 | 7 | 0 | 0 |
| 2000 | 7 | 3 | 0 | 0 | 0 | 0 | 0 | 0 | 0 | 0 |
| 2000 | 7 | 7 | 0 | 0 | 0 | 0 | 0 | 0 | 0 | 0 |
| 2000 | 7 | 11 | 1 | 0 | 0 | 0 | 14 | 4 | 0 | 0 |
| 2000 | 7 | 15 | 0 | 0 | 0 | 0 | 0 | 0 | 0 | 0 |
| 2000 | 7 | 19 | 1 | 1 | 0 | 0 | 5 | 0 | 0 | 0 |
| 2000 | 7 | 23 | 0 | 0 | 0 | 0 | 0 | 0 | 0 | 0 |
| 2000 | 7 | 27 | 0 | 0 | 0 | 0 | 0 | 0 | 0 | 0 |
| 2000 | 7 | 31 | 0 | 0 | 0 | 0 | 0 | 0 | 0 | 0 |
| 2001 | 5 | 12 | 0 | 0 | 0 | 0 | 0 | 0 | 0 | 0 |
| 2001 | 5 | 16 | 0 | 0 | 0 | 0 | 0 | 0 | 0 | 0 |
| 2001 | 5 | 20 | 0 | 0 | 0 | 0 | 0 | 0 | 0 | 0 |
| 2001 | 5 | 24 | 1 | 0 | 0 | 0 | 0 | 0 | 0 | 1 |
| 2001 | 5 | 28 | 3 | 1 | 0 | 0 | 0 | 0 | 2 | 0 |
| 2001 | 6 | 1 | 0 | 0 | 0 | 0 | 0 | 0 | 0 | 0 |
| 2001 | 6 | 5 | 5 | 1 | 0 | 0 | 0 | 0 | 0 | 0 |
| 2001 | 6 | 9 | 5 | 6 | 0 | 0 | 0 | 0 | 0 | 0 |
| 2001 | 6 | 13 | 0 | 0 | 0 | 0 | 0 | 0 | 0 | 0 |
| 2001 | 6 | 17 | 4 | 2 | 0 | 0 | 0 | 1 | 0 | 0 |
| 2001 | 6 | 21 | 0 | 0 | 0 | 0 | 0 | 0 | 0 | 0 |
| 2001 | 6 | 25 | 0 | 0 | 0 | 0 | 1 | 0 | 0 | 0 |
| 2001 | 6 | 29 | 0 | 0 | 0 | 0 | 0 | 0 | 0 | 0 |
| 2001 | 7 | 3 | 1 | 0 | 0 | 0 | 0 | 0 | 0 | 0 |
| 2001 | 7 | 7 | 0 | 0 | 0 | 0 | 0 | 0 | 0 | 0 |
| 2001 | 7 | 11 | 0 | 0 | 0 | 0 | 0 | 0 | 0 | 0 |
| 2001 | 7 | 15 | 0 | 0 | 0 | 0 | 0 | 0 | 0 | 0 |
| 2001 | 7 | 19 | 0 | 0 | 0 | 0 | 0 | 1 | 0 | 0 |
| 2001 | 7 | 23 | 0 | 0 | 0 | 0 | 0 | 0 | 0 | 0 |
| 2001 | 7 | 27 | 0 | 0 | 0 | 0 | 0 | 0 | 0 | 0 |
| 2002 | 5 | 12 | 0 | 0 | 0 | 0 | 0 | 0 | 0 | 0 |
| 2002 | 5 | 16 | 0 | 0 | 0 | 0 | 0 | 0 | 0 | 0 |
| 2002 | 5 | 20 | 0 | 0 | 0 | 0 | 0 | 0 | 0 | 0 |
| 2002 | 5 | 24 | 1 | 0 | 0 | 0 | 0 | 0 | 0 | 0 |
| 2002 | 5 | 28 | 0 | 0 | 0 | 0 | 0 | 0 | 0 | 0 |
| 2002 | 6 | 1 | 3 | 2 | 0 | 0 | 0 | 0 | 0 | 1 |
| 2002 | 6 | 5 | 20 | 16 | 0 | 0 | 0 | 0 | 1 | 0 |
| 2002 | 6 | 9 | 0 | 0 | 0 | 0 | 0 | 0 | 0 | 0 |
| 2002 | 6 | 13 | 2 | 6 | 0 | 0 | 0 | 1 | 0 | 0 |
| 2002 | 6 | 17 | 1 | 1 | 0 | 0 | 2 | 0 | 0 | 0 |
| 2002 | 6 | 21 | 0 | 0 | 0 | 0 | 0 | 0 | 0 | 0 |
| 2002 | 6 | 25 | 1 | 0 | 0 | 0 | 0 | 0 | 0 | 0 |
| 2002 | 6 | 29 | 0 | 0 | 0 | 0 | 0 | 0 | 0 | 0 |
| 2002 | 7 | 3 | 0 | 0 | 0 | 0 | 0 | 0 | 0 | 0 |
| 2002 | 7 | 7 | 0 | 0 | 0 | 0 | 0 | 0 | 0 | 0 |
| 2002 | 7 | 11 | 0 | 0 | 0 | 0 | 0 | 0 | 0 | 0 |
| 2002 | 7 | 15 | 0 | 0 | 0 | 0 | 0 | 0 | 0 | 0 |
| 2002 | 7 | 19 | 0 | 0 | 0 | 0 | 0 | 0 | 0 | 0 |
| 2002 | 7 | 23 | 0 | 0 | 0 | 0 | 0 | 0 | 0 | 0 |
| 2002 | 7 | 27 | 0 | 0 | 0 | 0 | 0 | 0 | 0 | 0 |
| 2003 | 5 | 12 | 0 | 0 | 0 | 0 | 0 | 0 | 0 | 0 |
| 2003 | 5 | 16 | 0 | 1 | 0 | 0 | 0 | 0 | 0 | 0 |
| 2003 | 5 | 20 | 0 | 0 | 0 | 0 | 0 | 0 | 0 | 0 |
| 2003 | 5 | 24 | 0 | 0 | 0 | 0 | 0 | 0 | 0 | 0 |
| 2003 | 5 | 28 | 3 | 2 | 0 | 0 | 0 | 0 | 0 | 1 |
| 2003 | 6 | 1 | 30 | 7 | 0 | 0 | 0 | 0 | 1 | 0 |
| 2003 | 6 | 5 | 0 | 0 | 0 | 0 | 0 | 0 | 0 | 0 |
| 2003 | 6 | 9 | 0 | 0 | 0 | 0 | 0 | 0 | 0 | 0 |
| 2003 | 6 | 13 | 9 | 2 | 0 | 0 | 1 | 0 | 0 | 0 |
| 2003 | 6 | 17 | 0 | 0 | 0 | 0 | 0 | 0 | 0 | 0 |
| 2003 | 6 | 21 | 2 | 0 | 0 | 0 | 0 | 1 | 0 | 0 |
| 2003 | 6 | 25 | 1 | 0 | 0 | 0 | 0 | 0 | 0 | 0 |
| 2003 | 6 | 29 | 0 | 0 | 0 | 0 | 0 | 0 | 0 | 0 |
| 2003 | 7 | 3 | 0 | 0 | 0 | 0 | 0 | 0 | 0 | 0 |
| 2003 | 7 | 7 | 0 | 0 | 0 | 0 | 0 | 0 | 0 | 0 |
| 2003 | 7 | 11 | 0 | 0 | 0 | 0 | 0 | 0 | 0 | 0 |
| 2003 | 7 | 15 | 0 | 0 | 0 | 0 | 0 | 0 | 0 | 0 |
| 2003 | 7 | 19 | 0 | 0 | 0 | 0 | 0 | 0 | 0 | 0 |
| 2003 | 7 | 23 | 0 | 0 | 0 | 0 | 0 | 0 | 0 | 0 |
| 2003 | 7 | 27 | 0 | 0 | 0 | 0 | 0 | 0 | 0 | 0 |
| 2004 | 5 | 12 | 0 | 0 | 0 | 0 | 0 | 0 | 0 | 0 |
| 2004 | 5 | 16 | 0 | 0 | 0 | 0 | 0 | 0 | 0 | 0 |
| 2004 | 5 | 20 | 1 | 0 | 0 | 0 | 0 | 0 | 0 | 0 |
| 2004 | 5 | 24 | 0 | 0 | 0 | 0 | 0 | 0 | 0 | 0 |
| 2004 | 5 | 28 | 3 | 1 | 0 | 0 | 0 | 0 | 0 | 0 |
| 2004 | 6 | 1 | 4 | 3 | 0 | 0 | 0 | 0 | 0 | 0 |
| 2004 | 6 | 5 | 0 | 0 | 0 | 0 | 0 | 0 | 0 | 0 |
| 2004 | 6 | 9 | 2 | 3 | 0 | 0 | 0 | 0 | 0 | 0 |
| 2004 | 6 | 13 | 0 | 0 | 0 | 0 | 0 | 0 | 0 | 0 |
| 2004 | 6 | 17 | 1 | 1 | 0 | 0 | 0 | 0 | 0 | 0 |
| 2004 | 6 | 21 | 0 | 0 | 0 | 0 | 0 | 0 | 0 | 0 |
| 2004 | 6 | 25 | 1 | 0 | 0 | 0 | 0 | 0 | 0 | 0 |
| 2004 | 6 | 29 | 0 | 0 | 0 | 0 | 0 | 0 | 0 | 0 |
| 2004 | 7 | 3 | 0 | 0 | 0 | 0 | 0 | 0 | 0 | 0 |
| 2004 | 7 | 7 | 0 | 0 | 0 | 0 | 0 | 0 | 0 | 0 |
| 2004 | 7 | 11 | 0 | 0 | 0 | 0 | 0 | 0 | 0 | 0 |
| 2004 | 7 | 15 | 0 | 0 | 0 | 0 | 0 | 0 | 0 | 0 |
| 2004 | 7 | 19 | 0 | 0 | 0 | 0 | 0 | 0 | 0 | 0 |
| 2004 | 7 | 23 | 0 | 0 | 0 | 0 | 0 | 0 | 0 | 0 |
| 2004 | 7 | 27 | 0 | 0 | 0 | 0 | 0 | 0 | 0 | 0 |
| 2005 | 5 | 12 | 0 | 0 | 0 | 0 | 0 | 0 | 0 | 0 |
| 2005 | 5 | 16 | 0 | 0 | 0 | 0 | 0 | 0 | 0 | 0 |
| 2005 | 5 | 20 | 0 | 0 | 0 | 0 | 0 | 0 | 0 | 0 |
| 2005 | 5 | 24 | 46 | 25 | 0 | 0 | 0 | 0 | 2 | 5 |
| 2005 | 5 | 28 | 42 | 49 | 0 | 0 | 0 | 0 | 8 | 13 |
| 2005 | 6 | 1 | 52 | 20 | 0 | 0 | 0 | 0 | 19 | 9 |
| 2005 | 6 | 5 | 0 | 0 | 0 | 0 | 0 | 0 | 0 | 0 |
| 2005 | 6 | 9 | 40 | 28 | 0 | 0 | 0 | 0 | 7 | 0 |
| 2005 | 6 | 13 | 25 | 6 | 0 | 0 | 0 | 0 | 0 | 3 |
| 2005 | 6 | 17 | 0 | 0 | 0 | 0 | 0 | 0 | 0 | 0 |
| 2005 | 6 | 21 | 6 | 11 | 0 | 0 | 16 | 16 | 0 | 0 |
| 2005 | 6 | 25 | 3 | 3 | 0 | 0 | 12 | 13 | 0 | 0 |
| 2005 | 6 | 29 | 0 | 0 | 0 | 0 | 0 | 0 | 0 | 0 |
| 2005 | 7 | 3 | 0 | 0 | 0 | 0 | 0 | 0 | 0 | 0 |
| 2005 | 7 | 7 | 1 | 0 | 0 | 0 | 8 | 5 | 0 | 0 |
| 2005 | 7 | 11 | 3 | 0 | 0 | 0 | 2 | 0 | 0 | 0 |
| 2005 | 7 | 15 | 0 | 0 | 0 | 0 | 0 | 0 | 0 | 0 |
| 2005 | 7 | 19 | 0 | 0 | 0 | 0 | 0 | 0 | 0 | 0 |
| 2005 | 7 | 23 | 0 | 0 | 0 | 0 | 0 | 0 | 0 | 0 |
| 2005 | 7 | 27 | 0 | 0 | 0 | 0 | 0 | 0 | 0 | 0 |
| 2005 | 7 | 31 | 0 | 0 | 0 | 0 | 0 | 0 | 0 | 0 |
| 2006 | 5 | 29 | 83 | 43 | 0 | 2 | 0 | 0 | 3 | 11 |
| 2006 | 6 | 2 | 88 | 47 | 1 | 2 | 0 | 0 | 21 | 18 |
| 2006 | 6 | 7 | 71 | 59 | 2 | 1 | 0 | 0 | 9 | 0 |
| 2006 | 6 | 12 | 77 | 41 | 0 | 0 | 2 | 1 | 0 | 0 |
| 2006 | 6 | 14 | 11 | 15 | 0 | 1 | 6 | 2 | 0 | 0 |
| 2006 | 6 | 20 | 45 | 34 | 0 | 0 | 12 | 15 | 0 | 0 |
| 2006 | 6 | 26 | 7 | 2 | 0 | 0 | 16 | 12 | 0 | 0 |
| 2006 | 7 | 3 | 2 | 3 | 0 | 0 | 16 | 13 | 0 | 0 |
| 2006 | 7 | 7 | 5 | 0 | 0 | 0 | 9 | 11 | 0 | 0 |
| 2006 | 7 | 13 | 1 | 1 | 0 | 0 | 11 | 4 | 0 | 0 |
| 2007 | 5 | 10 | 0 | 0 | 0 | 1 | 0 | 0 | 0 | 0 |
| 2007 | 5 | 14 | 0 | 3 | 0 | 0 | 0 | 0 | 0 | 0 |
| 2007 | 5 | 17 | 9 | 6 | 0 | 0 | 0 | 0 | 0 | 0 |
| 2007 | 5 | 21 | 35 | 25 | 1 | 0 | 0 | 0 | 2 | 8 |
| 2007 | 5 | 24 | 54 | 28 | 3 | 4 | 0 | 0 | 5 | 11 |
| 2007 | 5 | 28 | 74 | 60 | 12 | 4 | 0 | 0 | 18 | 5 |
| 2007 | 5 | 31 | 9 | 0 | 0 | 0 | 0 | 0 | 0 | 0 |
| 2007 | 6 | 4 | 105 | 57 | 2 | 1 | 0 | 0 | 7 | 5 |
| 2007 | 6 | 7 | 76 | 62 | 1 | 0 | 1 | 0 | 3 | 1 |
| 2007 | 6 | 11 | 67 | 26 | 0 | 0 | 0 | 0 | 0 | 0 |
| 2007 | 6 | 14 | 35 | 15 | 1 | 0 | 3 | 2 | 0 | 0 |
| 2007 | 6 | 18 | 28 | 27 | 0 | 0 | 3 | 2 | 0 | 0 |
| 2007 | 6 | 21 | 21 | 15 | 0 | 0 | 1 | 1 | 0 | 0 |
| 2007 | 6 | 25 | 5 | 0 | 0 | 0 | 1 | 3 | 0 | 0 |
| 2007 | 6 | 28 | 11 | 0 | 0 | 0 | 18 | 4 | 0 | 0 |
| 2007 | 7 | 2 | 1 | 0 | 0 | 0 | 6 | 2 | 0 | 0 |
| 2007 | 7 | 5 | 0 | 1 | 0 | 0 | 5 | 0 | 0 | 0 |
| 2007 | 7 | 9 | 10 | 2 | 0 | 0 | 3 | 1 | 0 | 0 |
| 2007 | 7 | 12 | 1 | 3 | 0 | 0 | 3 | 0 | 0 | 0 |
| 2007 | 7 | 16 | 1 | 0 | 0 | 0 | 0 | 1 | 0 | 0 |
| 2007 | 7 | 19 | 0 | 0 | 0 | 0 | 0 | 0 | 0 | 0 |
| 2007 | 7 | 23 | 1 | 0 | 0 | 0 | 0 | 0 | 0 | 0 |
| 2007 | 7 | 26 | 2 | 2 | 0 | 0 | 0 | 0 | 0 | 0 |
| 2007 | 7 | 30 | 3 | 2 | 0 | 0 | 0 | 0 | 0 | 0 |
| 2008 | 5 | 16 | 0 | 0 | 0 | 0 | 0 | 0 | 0 | 0 |
| 2008 | 5 | 19 | 0 | 0 | 0 | 0 | 0 | 0 | 0 | 0 |
| 2008 | 5 | 22 | 7 | 3 | 0 | 0 | 0 | 0 | 3 | 4 |
| 2008 | 5 | 25 | 67 | 24 | 0 | 0 | 0 | 0 | 10 | 33 |
| 2008 | 5 | 29 | 49 | 20 | 0 | 2 | 0 | 0 | 23 | 11 |
| 2008 | 6 | 1 | 96 | 71 | 29 | 35 | 0 | 0 | 70 | 19 |
| 2008 | 6 | 4 | 16 | 21 | 30 | 23 | 0 | 0 | 27 | 12 |
| 2008 | 6 | 7 | 75 | 68 | 11 | 8 | 0 | 0 | 4 | 2 |
| 2008 | 6 | 10 | 80 | 52 | 1 | 0 | 0 | 0 | 5 | 1 |
| 2008 | 6 | 13 | 68 | 45 | 2 | 0 | 0 | 0 | 0 | 0 |
| 2008 | 6 | 16 | 40 | 27 | 1 | 1 | 0 | 4 | 0 | 0 |
| 2008 | 6 | 19 | 31 | 12 | 0 | 1 | 1 | 3 | 0 | 0 |
| 2008 | 6 | 22 | 45 | 28 | 0 | 0 | 6 | 4 | 0 | 0 |
| 2008 | 6 | 25 | 32 | 20 | 0 | 0 | 4 | 12 | 0 | 0 |
| 2008 | 6 | 30 | 5 | 1 | 0 | 0 | 3 | 1 | 0 | 0 |
| 2008 | 7 | 3 | 8 | 13 | 0 | 0 | 17 | 13 | 0 | 0 |
| 2008 | 7 | 6 | 13 | 3 | 0 | 0 | 20 | 14 | 0 | 0 |
| 2008 | 7 | 10 | 9 | 8 | 0 | 0 | 18 | 15 | 0 | 0 |
| 2008 | 7 | 14 | 4 | 0 | 0 | 0 | 2 | 0 | 0 | 0 |
| 2008 | 7 | 18 | 3 | 4 | 0 | 0 | 0 | 0 | 0 | 0 |
| 2008 | 7 | 22 | 1 | 1 | 0 | 0 | 7 | 6 | 0 | 0 |
| 2008 | 7 | 26 | 8 | 1 | 0 | 0 | 1 | 0 | 0 | 0 |
| 2008 | 7 | 30 | 0 | 0 | 0 | 0 | 0 | 0 | 0 | 0 |
| 2009 | 5 | 10 | 0 | 0 | 0 | 0 | 0 | 0 | 0 | 0 |
| 2009 | 5 | 11 | 0 | 0 | 0 | 0 | 0 | 0 | 0 | 0 |
| 2009 | 5 | 14 | 0 | 3 | 0 | 0 | 0 | 0 | 0 | 0 |
| 2009 | 5 | 18 | 0 | 0 | 0 | 0 | 0 | 0 | 0 | 0 |
| 2009 | 5 | 22 | 0 | 1 | 0 | 2 | 0 | 0 | 1 | 1 |
| 2009 | 5 | 25 | 6 | 11 | 10 | 34 | 0 | 0 | 17 | 52 |
| 2009 | 5 | 28 | 22 | 31 | 31 | 32 | 0 | 0 | 130 | 118 |
| 2009 | 6 | 1 | 82 | 94 | 19 | 16 | 0 | 0 | 32 | 17 |
| 2009 | 6 | 4 | 111 | 97 | 4 | 9 | 0 | 0 | 20 | 8 |
| 2009 | 6 | 8 | 107 | 97 | 3 | 0 | 0 | 0 | 8 | 0 |
| 2009 | 6 | 11 | 119 | 84 | 1 | 0 | 0 | 0 | 7 | 1 |
| 2009 | 6 | 15 | 66 | 46 | 1 | 0 | 0 | 0 | 13 | 7 |
| 2009 | 6 | 19 | 50 | 31 | 0 | 0 | 0 | 0 | 16 | 15 |
| 2009 | 6 | 22 | 5 | 1 | 0 | 0 | 0 | 0 | 0 | 0 |
| 2009 | 6 | 30 | 4 | 0 | 0 | 0 | 10 | 15 | 0 | 0 |
| 2009 | 7 | 3 | 14 | 8 | 0 | 0 | 34 | 19 | 0 | 0 |
| 2009 | 7 | 10 | 0 | 0 | 0 | 0 | 9 | 12 | 0 | 0 |
| 2009 | 7 | 17 | 2 | 0 | 0 | 0 | 0 | 0 | 0 | 0 |
| 2009 | 7 | 22 | 1 | 0 | 0 | 0 | 1 | 1 | 0 | 0 |
| 2009 | 7 | 30 | 0 | 0 | 0 | 0 | 0 | 0 | 0 | 0 |
| 2010 | 5 | 11 | 0 | 0 | 0 | 0 | 0 | 0 | 0 | 0 |
| 2010 | 5 | 14 | 0 | 0 | 0 | 0 | 0 | 0 | 0 | 0 |
| 2010 | 5 | 17 | 0 | 0 | 0 | 0 | 0 | 0 | 0 | 0 |
| 2010 | 5 | 20 | 0 | 0 | 0 | 0 | 0 | 0 | 0 | 0 |
| 2010 | 5 | 24 | 0 | 0 | 0 | 0 | 0 | 0 | 0 | 0 |
| 2010 | 5 | 28 | 1 | 4 | 0 | 0 | 0 | 0 | 1 | 6 |
| 2010 | 6 | 4 | 23 | 13 | 0 | 0 | 0 | 0 | 7 | 6 |
| 2010 | 6 | 7 | 16 | 19 | 2 | 4 | 0 | 0 | 29 | 16 |
| 2010 | 6 | 10 | 13 | 8 | 4 | 4 | 0 | 0 | 4 | 6 |
| 2010 | 6 | 13 | 18 | 24 | 6 | 8 | 0 | 0 | 11 | 7 |
| 2010 | 6 | 16 | 8 | 4 | 2 | 2 | 0 | 0 | 1 | 0 |
| 2010 | 6 | 20 | 13 | 9 | 0 | 0 | 0 | 0 | 4 | 1 |
| 2010 | 6 | 22 | 4 | 12 | 0 | 0 | 1 | 3 | 0 | 0 |
| 2010 | 6 | 25 | 5 | 4 | 0 | 0 | 0 | 1 | 0 | 0 |
| 2010 | 6 | 30 | 9 | 5 | 0 | 0 | 1 | 0 | 0 | 0 |
| 2010 | 7 | 1 | 0 | 0 | 0 | 0 | 0 | 1 | 0 | 0 |
| 2010 | 7 | 15 | 0 | 0 | 0 | 0 | 0 | 1 | 0 | 0 |
| 2010 | 7 | 20 | 0 | 0 | 0 | 0 | 0 | 0 | 0 | 0 |
