# Supplementary material for: Structural dynamics in the host-parasitoid system of the pine needle gall midge (Thecodiplosis japonensis) during invasion
Source: PeerJ. 2017 Aug 22;5:e3610. doi: 10.7717/peerj.3610 (PMC5571814; doi:10.7717/peerj.3610)
Supplement: Table S2 — Changes of meteorological factors measured at Yeongcheon weather station near the study site during the study period 1986–2010. Data were obtained from the Korean Meteorological Administration (KMA; http://www.kma.go.kr). Minimum temperature: mean of daily minimum temperature in January, maximum temperature: mean of daily maximum temperature in July and August, mean temperature: annual mean temperature, and precipitation: precipitation in spring from March to May. [file peerj-05-3610-s002.docx]

Table S2. Changes of meteorological factors measured at Yeongcheon weather station near the study site during the study period 1986 - 2010. Data were obtained from the Korean Meteorological Administration (KMA; <http://www.kma.go.kr>). Minimum temperature: mean of daily minimum temperature in January, maximum temperature: mean of daily maximum temperature in July and August, mean temperature: annual mean temperature, and precipitation: precipitation in spring from March to May.

| Year | Precipitation (mm) | Maximum temperature (℃) | Minimum temperature (℃) | Mean temperature (℃) |
| --- | --- | --- | --- | --- |
| 1986 | 163.5 | 29.1 | -7.2 | 11.6 |
| 1987 | 142.9 | 28.9 | -4.5 | 12.0 |
| 1988 | 133.9 | 28.8 | -5.9 | 11.8 |
| 1989 | 214.3 | 28.3 | -2.6 | 12.5 |
| 1990 | 162.6 | 31.6 | -3.4 | 13.0 |
| 1991 | 214.3 | 27.3 | -5.9 | 11.9 |
| 1992 | 250.6 | 29.7 | -4.5 | 12.1 |
| 1993 | 246.8 | 26.4 | -5.6 | 11.5 |
| 1994 | 170.3 | 34.3 | -5.6 | 13.4 |
| 1995 | 185.5 | 31.8 | -5.8 | 12.2 |
| 1996 | 250.1 | 30.6 | -6.5 | 12.0 |
| 1997 | 182.5 | 30.0 | -7.0 | 12.5 |
| 1998 | 240.5 | 28.5 | -4.5 | 13.2 |
| 1999 | 262.0 | 27.7 | -5.1 | 12.4 |
| 2000 | 117.0 | 31.2 | -5.4 | 12.8 |
| 2001 | 75.0 | 31.9 | -5.5 | 12.8 |
| 2002 | 233.0 | 28.9 | -4.4 | 12.5 |
| 2003 | 384.5 | 27.3 | -5.7 | 12.3 |
| 2004 | 185.3 | 30.9 | -5.5 | 13.2 |
| 2005 | 171.7 | 30.5 | -5.2 | 12.5 |
| 2006 | 280.4 | 30.4 | -4.8 | 12.7 |
| 2007 | 181.5 | 30.1 | -4.0 | 13.1 |
| 2008 | 163.5 | 30.8 | -5.3 | 12.7 |
| 2009 | 166.1 | 27.9 | -4.8 | 12.7 |
| 2010 | 235.4 | 31.4 | -5.2 | 12.4 |
